# Effectiveness of long-term using statins in COPD – a network meta-analysis

**DOI:** 10.1186/s12931-019-0984-3

**Published:** 2019-01-23

**Authors:** Yongbin Lu, Ruixia Chang, Jia Yao, Xinni Xu, Yongjun Teng, Ning Cheng

**Affiliations:** 1 The First Hospital of Lanzhou University, Lanzhou, Gansu People’s Republic of China; 2 Lanzhou Maternal and Child Health Care Hospital, Lanzhou, Gansu 730000 People’s Republic of China; 3 Lanzhou University, Basic Medical College, Lanzhou, Gansu 730000 People’s Republic of China

**Keywords:** Statins, Mortality, CRP, PH, Network meta-analysis

## Abstract

**Objectives:**

To evaluate the effectiveness of long-term treatment of statins for chronic obstructive pulmonary disease (COPD), and to answer which one is better.

**Methods:**

General meta-analysis was performed to produce polled estimates of the effect of mortality, inflammatory factors, and lung function index in COPD patients by the search of PubMed, Web of Science, Embase, and China National Knowledge Infrastructure for eligible studies. A network meta-analysis was performed to synthetically compare the effectiveness of using different statins in COPD patients.

**Results:**

General meta-analysis showed that using statins reduced the risk of all-cause mortality, heart disease-related mortality and COPD acute exacerbation (AECOPD) in COPD patients, the RR (95% CI) were 0.72 (0.63,0.84), 0.72 (0.53,0.98) and 0.84 (0.79,0.89), respectively. And using statins reduced C-reactive protein (CRP) and pulmonary hypertension (PH) in COPD patients, the SMD (95% CI) were − 0.62 (− 0.52,-0.72) and − 0.71 (− 0.85,-0.57), respectively. Network meta-analysis showed that Fluvastatin (97.7%), Atorvastatin (68.0%) and Rosuvastatin (49.3%) had higher cumulative probability than other statins in reducing CRP in COPD patients. Fluvastatin (76.0%) and Atorvastatin (75.4%) had higher cumulative probability than other satins in reducing PH in COPD patients.

**Conclusions:**

Using statins can reduce the risk of mortality, the level of CRP and PH in COPD patients. In addition, Fluvastatin and Atorvastatin are more effective in reducing CRP and PH in COPD patients.

**Electronic supplementary material:**

The online version of this article (10.1186/s12931-019-0984-3) contains supplementary material, which is available to authorized users.

## Backgrounds

Chronic obstructive pulmonary disease (COPD) affects 380 million people worldwide, representing 12% of adults over 30 years of age, the prevalence of this disease is rapidly increasing, and it will be the third cause of mortality worldwide in 2020 [1, 2]. Comorbidities associated with COPD are important aspect of the disease; in particular, cardiovascular disease (CVD) has been shown to be greater than 2 times more prevalent in COPD patients compared with normal population [3, 4]. What’s more, pulmonary hypertension (PH) is one of the most important functional derangements in COPD and leads poor prognosis, which mechanisms have been ascribed to chronic hypoxia and loss of vascular bed from emphysematous destruction [5, 6]. The importance of CVD and other comorbidities in COPD patients is such that all-cause mortality has largely become the most relevant metric for outcomes in COPD patients [7]. Localized chronic inflammation of the airways has long been observed in COPD patients, and there is a growing understanding of systemic inflammation in subset of COPD patients [8, 9]. Specifically, high levels of CRP and IL-6 have been associated with poor outcomes in COPD patients [10, 11].

Statins are widely used for treatment of hypercholesterolemia and CVD, as a class of cholesterol lowering drugs. In addition to their well-established benefits for CVD, they have also been studied in a variety of other disease states, including dementia, contrast-induced nephropathy, and erectile dysfunction and COPD in most notably over the past decade [12, 13]. What needs to be emphasized is that anti-inflammatory actions modulate the immune system and reduce inflammatory markers such as CRP and IL-6 are slowly being discovered [11, 14]. Systematic reviews prior to 2017 have been suggested that statins are associated with a beneficial role in treatment of COPD [15–17], which including reduce all-cause mortality, cause-specific mortality and PH. Li et al. research which including twenty studies with a total of 303,981 patients found [16] statins reduced all-cause mortality and COPD exacerbation, but not to hospitalization. Zhang et al. which including 6 RCTS with both COPD and PH patients found statin therapy was associated with increased 6-min walk test (6 MW) and reduced PH, but there was no observed difference in all-cause mortality [17]. Whether statins can reduce inflammation and PH or not remains controversial [17, 18].

Previous meta-analysis of statin involved more all-cause mortality and fewer inflammatory factors. Even if involved, it is a general proof that statins can reduce certain inflammatory mediators. Whether statins reduce inflammatory mediators and pulmonary hypertension in COPD patients, ultimately reducing patient mortality? Which statin is more effective in treating COPD patients? This network meta-analysis study the role of statins in the treatment of COPD which including short-term inflammatory mediators and long-term final outcome measures. Our network meta-analysis answers whether statins can reduce inflammation and PH, and which statin is more effective for COPD in the first time, and provide evidence for the clinical treatment of COPD.

## Materials and methods

### Search strategy

We searched PubMed, Web of Science, Embase, and China National Knowledge Infrastructure for eligible studies from January 1990 to March 2018, including studies ahead of publications and without language restriction. We also performed a manual search of references cited and related in the important studies. The combinations of keywords used were (“Pulmonary Disease, Chronic Obstructive” or “Chronic Obstructive Pulmonary Disease” or “COPD” or “Chronic Obstructive Airway Disease” or “COAD” or “Chronic Obstructive Lung Disease” or “Chronic Airflow Obstruction” or “Lung Diseases, Obstructive” or “Obstructive Lung Disease” or “Obstructive Pulmonary Disease” or “Lung Disease” or “Pulmonary Disease”) and (“Hydroxymethylglutaryl-CoA Reductase Inhibitors” or “HMG-CoA Reductase Inhibitors” or “Statin” or “Lovastatin” or “Pravastatin” or “Mevastatin” or “Fluvastatin” or “Atorvastatin” or “Lipitor” or “Rosuvastatin” or “pitavastatin” or “Simvastatin” or “Cerivastatin”).

### Study selection and quality evaluation

Studies were included in the meta-analysis if they met the following criteria: (1) Published cohort studies or randomized controlled trials (RCT); (2) Studies with ≥1 months of follow-up: (3) relative risk (RR), HR estimated with 95%CI or sufficient data to calculate were reported for binary variables; standardized mean difference (SMD) estimated with 95%CI or sufficient data to calculate were reported for continuous variables; (4) studies in which the number of events and total number of participants in each study group were reported; (5) the outcome of interest were long-term outcome indicators including all-cause and cause-specific mortality, and short-term outcome indicators including inflammatory factors of CRP, IL-6, Interleukin-8 (IL-8) and tumor necrosis factor-α (TNF-α), lung function index of forced expiratory volume in one second percent (FEV1%), forced expiratory volume in one second/forced vital capacity (FEV1/FVC%), 6 MW and PH (measured by color Doppler echocardiography to determine tricuspid regurgitation velocity, calculated according to Bernoulli’s formula (*P* = 4 V2)), blood lipids index of total cholesterol (TC) and triglyceride (TG); (6) Interventions must include two groups of statin and placebo. We excluded studies published as abstracts and review as we considered the information to be insufficient for our assessment.

Two authors independently extracted relevant information from different studies. The following data were extracted from each study: the first author’s name, publish year, research methods, area, treatment, participants age, follow-up duration, the number of events and total number of participants in the intervention and control groups and outcomes as far as reported. Disagreements in data extracted were resolved by consult.

We used the Newcastle-Ottawa quality assessment scale for quality assessment of cohort studies [19]. The scale is judged based on selection (four items), comparability (one item), and exposure/outcome (three items). We used the Cochrane Collaboration risk of bias tool assessment scale for quality assessment of RCT, which including appropriateness of allocation, blinding, and management of incomplete outcome data; completeness of reporting of outcomes and other bias [20].

### Sensitivity analysis and publication bias

Sensitivity analysis was performed to evaluate the Stability and reliability of the results, in which subgroup analysis was performed by statins. We compared the pooled RR and SMD estimates from different subgroups and the overall estimate. Publication bias was evaluated by Egger’s test. Forest plots and funnel plots were used to examine the overall effect and assess the publication bias, respectively.

### Statistical methods

Pairwise comparisons of each statin and effect versus control were performed by using random effect model (Stata 12.0). The random effects model is more powerful than the fixed effect model and incorporates into the weighing scheme both within-study and between-study variations [21]. We performed further subgroup analysis for inflammatory factors, lung function index and blood lipids index in the intervention and control groups. Statistical heterogeneity were evaluated by using Cochran Q statistic and quantified by *I*^*2*^ statistic [22]. When heterogeneity was present, sensitivity analysis and subgroup analysis were performed to identify responsible outlier studies.

We used the multiple treatment meta-analysis method (MTM) proposed by Salanti et al. (a Bayesian method based on the Markov Chain Monte Carlosimulation) to compare testing modalities for effect from statins [23]. All MTMs were performed using WinBugs version 1.4.3 utilizing random effects models. The estimates obtained by generating five chains with 1000 initial iterations (burn in) and 10,000 iterations were used for estimations. We assessed the probability that which statin is best by calculating its treatment effect compared with control and counting the proportion of iterations of the Markov chain in which each statin has the highest treatment effect, second highest, and so on. We developed rankograms and cumulative probability plots to graphically present the distribution of ranking probabilities and estimated the surface under the cumulative ranking (SUCRA) line for each statin [24].

## Results

### Characteristics of eligible studies

A total of 988 studies were identified in the original database search, of which 138 needed to be further screened. Finally, 53 studies were included (Additional file 1: Figure S1).

Of the 53 studies, 14 studies [11, 25–37] reported all-cause mortality, 4 studies [26, 30, 36, 38] reported heart disease-related mortality, 6 studies [27, 30, 34, 35, 38, 39] reported COPD mortality, 12 studies [11, 25, 28, 30, 38, 40–46] reported AECOPD, 20 studies [44, 46–64] reported CRP, 12 studies [44, 47, 50, 51, 54, 59, 62, 65–69] reported IL-6, 5 studies [55, 59, 60, 65, 70] reported IL-8, 9 studies [51, 53, 58–62, 65, 68] reported TNF-α, 17 studies [5, 45, 47, 49–52, 57, 63, 65, 67, 68, 71–75] reported FEV1%, 15 studies [5, 50, 51, 55, 59, 60, 65, 67, 68, 70–75] reported FEV1/FVC%, 7 studies [5, 52, 60–62, 66, 70] reported 6 MW, 9 studies [36, 47, 48, 51, 58, 66, 70, 71, 74] reported TC, 6 studies [47, 48, 66, 70, 71, 74] reported TG and 10 studies [5, 45, 52, 55, 56, 60, 63, 66, 73, 74] reported PH. The number of patients in each studies ranged from 40 to 68,754. The duration of follow-up ranged from one to 120 months (Table 1).

**Table 1:**
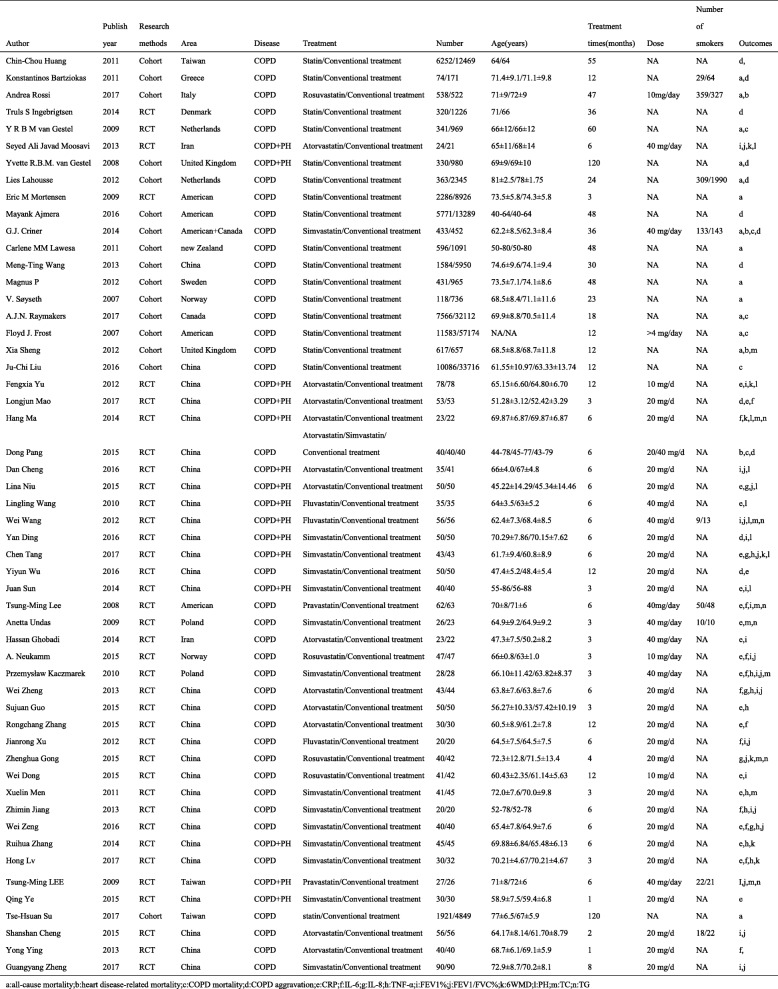
Study characteristic

a: all-cause mortality; b: heart disease-related mortality; c: COPD mortality; d: COPD aggravation; e:CRP; f:IL-6; g:IL-8;

h:TNF-α;i:FEV1%;j:FEV1/FVC%;k:6WMD;1:PH;m:TC;n:TG

### Using statins reduced the risk of all-cause mortality and cause-specific mortality

**Fig. 1:**
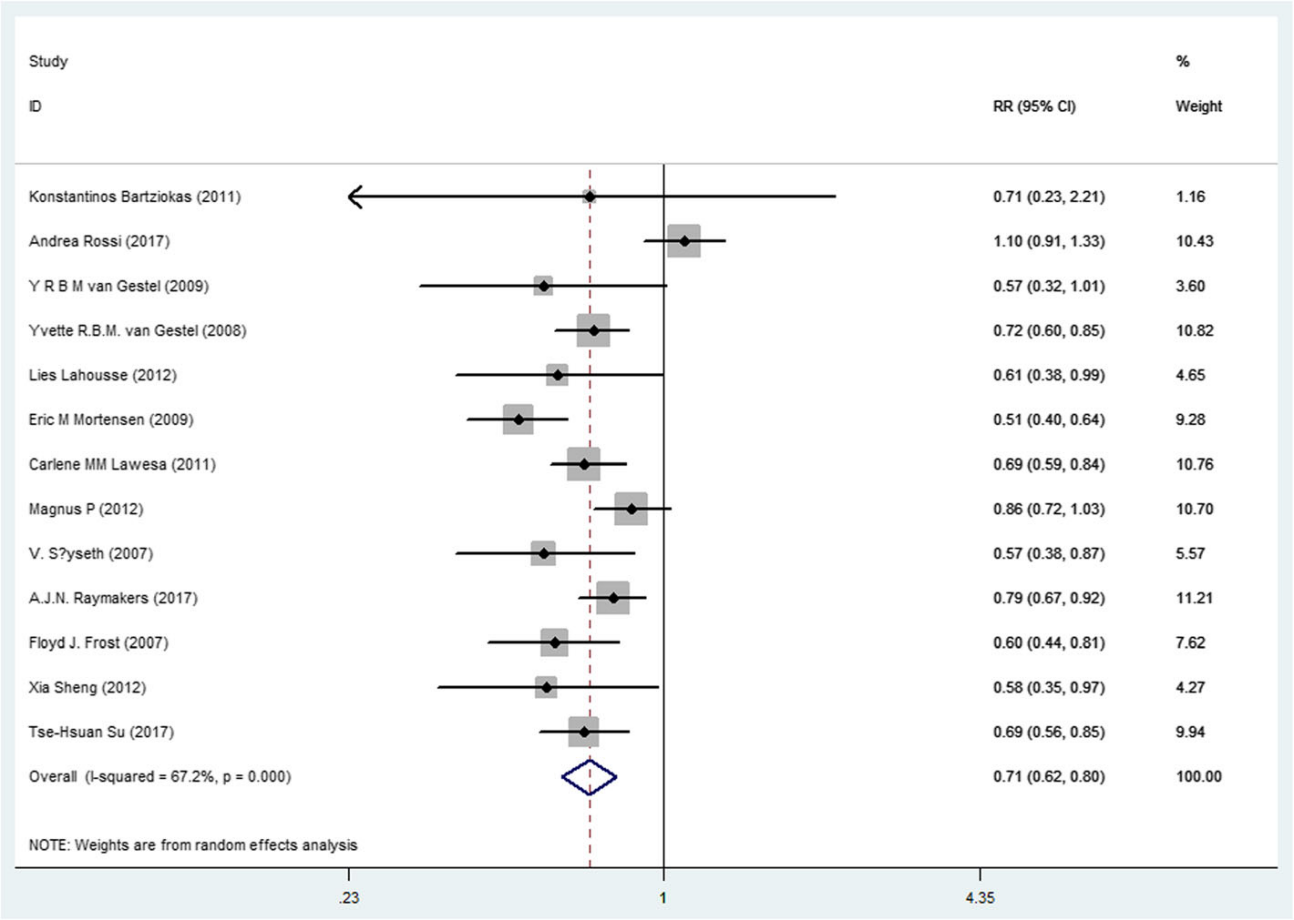
Forest plot showing effect of statins on all-cause mortality in COPD patients

Random effect models analyses showed that using statins significantly reduced the risk of all-cause mortality, the RR (95% CI) was 0.72 (0.63, 0.84), with a significant strong heterogeneity (*I*^*2*^ *= 86.8%, P < 0.01*). Figure 1 showed the heterogeneity reduced when using sensitivity analysis and exclude a study [30] (*I*^*2*^ *= 67.2%, P < 0.01*), the RR (95% CI) was 0.71 (0.62, 0.80). Using statins didn’t reduced the risk of heart disease-related mortality, the RR (95% CI) was 0.92 (0.83, 1.03) (Additional file 2: Figure S2). Using statins reduced the risk of COPD mortality and AECOPD, the RR (95% CI) were 0.72 (0.53, 0.98) and 0.84 (0.79, 0.89), respectively (Additional file 3: Figure S3 and Additional file 4: Figure S4).

### Using statins reduced the level of CRP, IL-6, IL-8 and TNF-α

**Fig. 2:**
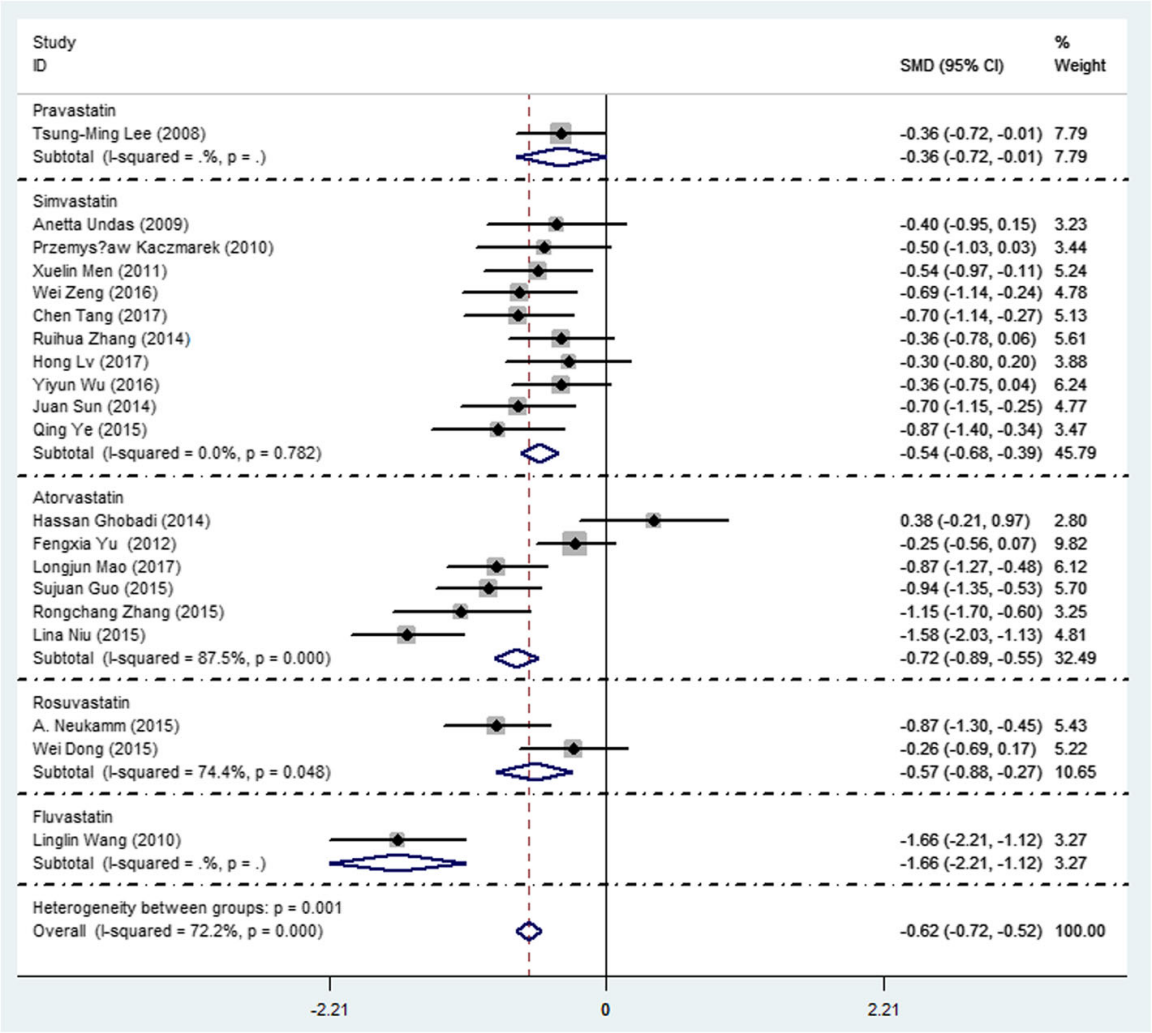
Forest plot showing effect of statins on CRP in COPD patients

**Fig. 3:**
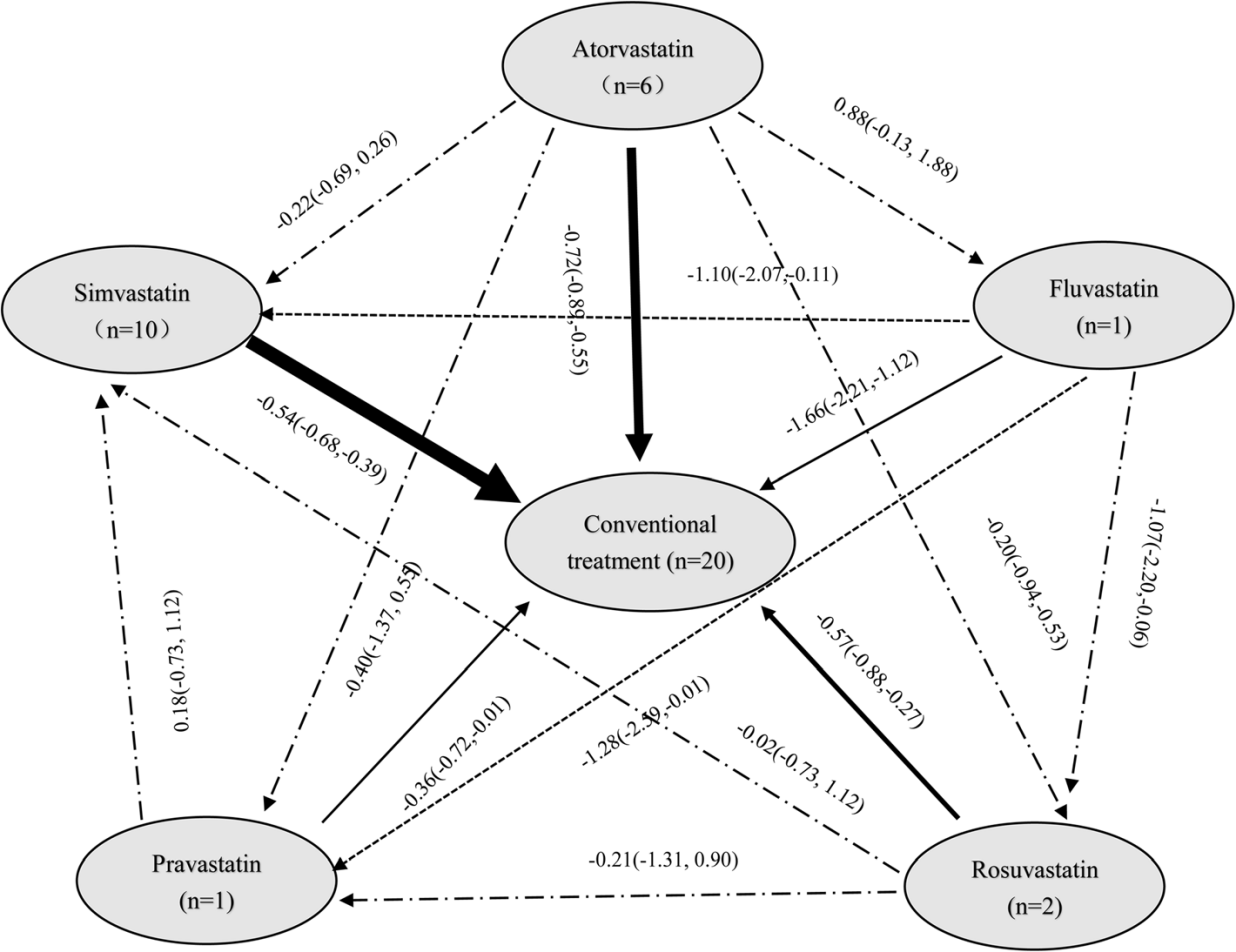
Evidence network of the effect of using statins on CRP levels in COPD patients

**Fig. 4:**
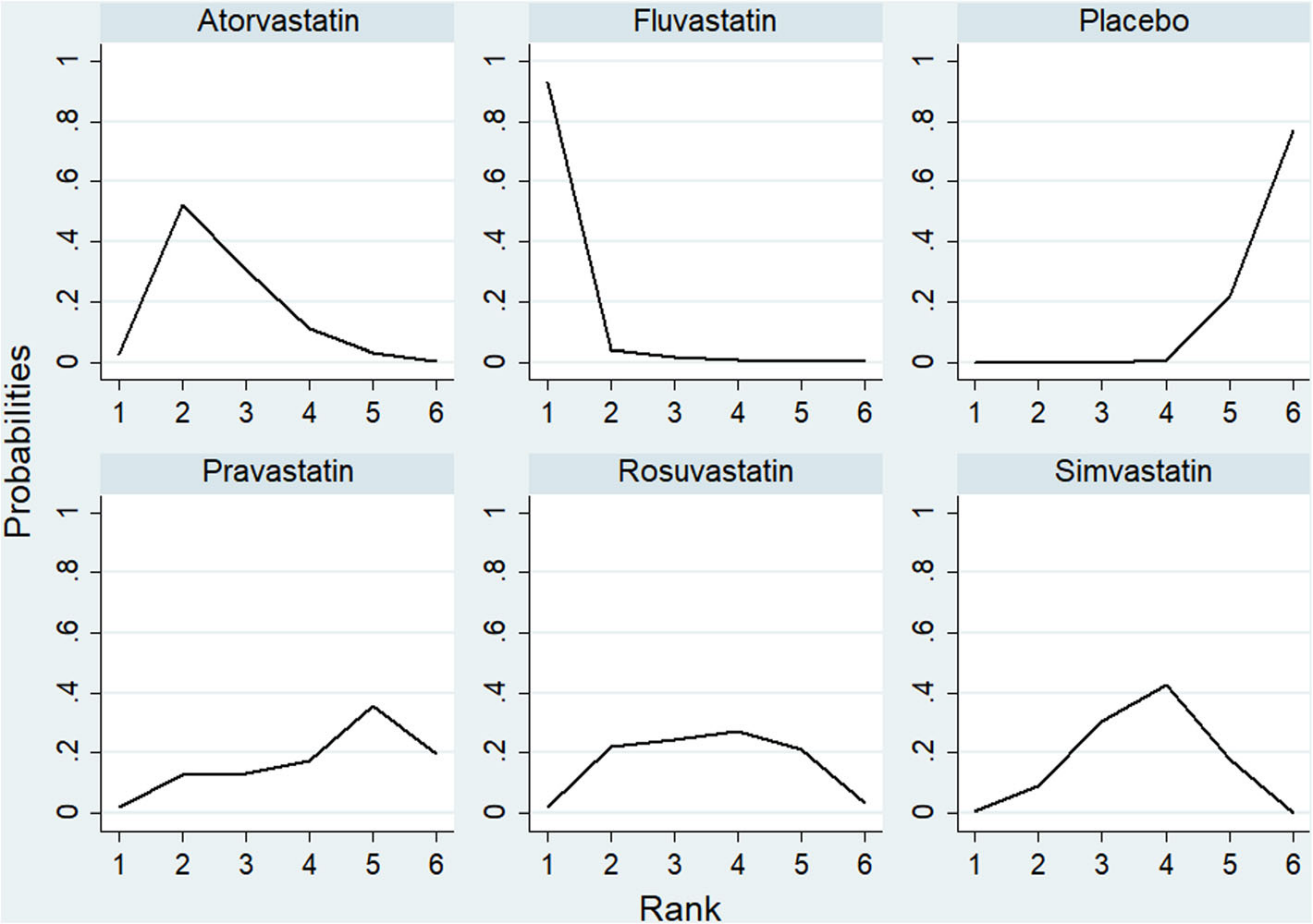
Rank probability analysis of CRP with using statins in COPD patients

Figure 2 showed the changes of CRP after using statins in COPD patients, which showed statins therapy significantly reduced CRP, the SMD (95% CI) was − 0.62 (− 0.52,-0.72). Subgroup analysis showed Pravastatin, Simvastatin, Atorvastatin, Rosuvastatin, Fluvastatin were significantly reduced CRP, the SMD (95% CI) were − 0.36 (− 0.72,-0.01), − 0.54 (− 0.68,-0.39), − 0.72 (− 0.89,-0.55), − 0.57 (− 0.88,-0.27) and − 1.66 (− 0.2.21,-1.12), respectively. Figure 3 showed the diagram of network meta-analysis between the SMD changes of CRP after using different statins in COPD patients. Network meta-analysis showed that Fluvastatin significantly reduced CRP compared Pravastatin and Simvastatin, the SMD (95% CI) were − 1.28 (− 2.59, − 0.01) and − 1.10 (− 2.07, − 0.11), respectively. The SUCRA of reducing the CRP in COPD patients in Atorvastatin, Fluvastatin, Rosuvastatin, Pravastatin and Simvastatin were 68.0, 97.7, 49.3, 33.9 and 46.3%, respectively (Table 2 and Fig. 4). Across all patients, Fluvastatin, Atorvastatin and Rosuvastatin had higher overall probability, and they were more effective in reducing CRP than other statins in COPD patients.

**Table 2:** Rank probability analysis of CRP with using stains in COPD patients

Treatment	SUCRA	sd	2.50%	median	97.50%
Atorvastatin	68.0	0.1685	0.2	0.8	1.0
Fluvastatin	97.7	0.0969	0.6	1.0	1.0
Rosuvastatin	49.3	0.2392	0.0	0.4	0.8
Pravastatin	33.9	0.2718	0.0	0.2	0.8
Simvastatin	46.3	0.1759	0.2	0.4	0.8
Conventional treatment	4.8	0.0900	0.0	0.0	0.2

**Table 3:** Rank probability analysis of PH with using stains in COPD patients

Treatment	SUCRA	sd	2.50%	median	97.50%
Atorvastatin	75.4	0.2354	0.3	0.7	1.0
Fluvastatin	76.0	0.2616	0.3	0.7	1.0
Simvastatin	48.0	0.2299	0.3	0.3	1.0
Conventional treatment	0.6	0.0431	0.0	0.0	0.0

**Fig. 5:**
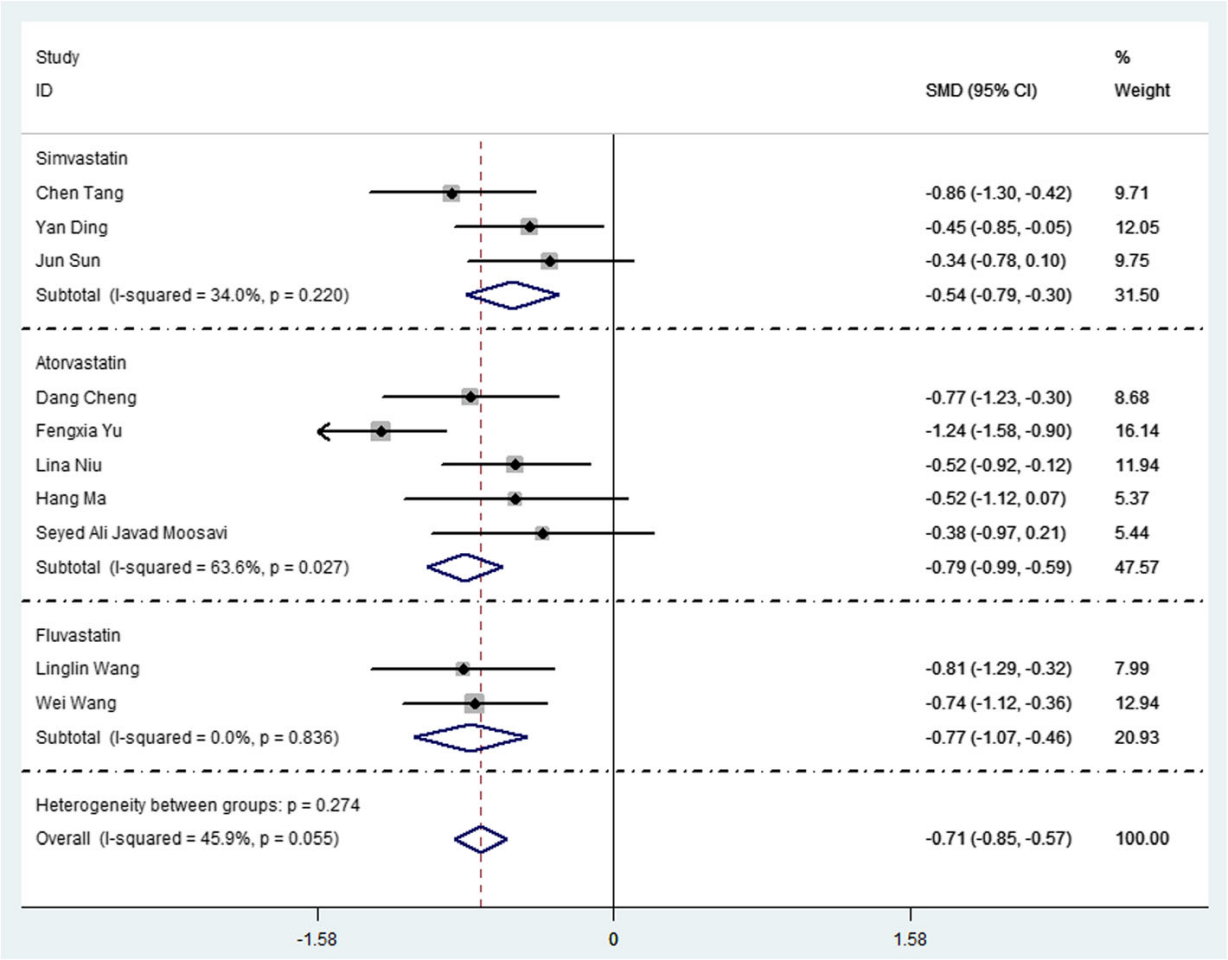
Forest plot showing effect of statins on PH in COPD patients

Additional file 5: Figure S5 showed that statins therapy significantly reduced IL-6, the SMD (95% CI) was − 0.95 (− 1.09,-0.81). Subgroup analysis showed Pravastatin (1 study), Rosuvastatin, Simvastatin and Atorvastatin were significantly reduced IL-6, and the SMD (95% CI) were − 1.00 (− 1.38,-0.63), − 1.34 (− 1.79,-0.89), − 1.25 (− 1.56,-0.95) and − 0.79 (− 1.01,-0.58), respectively. Additional file 6: Figure S6 showed the diagram of network meta-analysis between the SMD changes of IL-6 after using different statins in COPD patients. Network meta-analysis showed that the SUCRA of reducing the IL-6 in COPD patients in Atorvastatin, Fluvastatin, Rosuvastatin, Pravastatin and Simvastatin were 52.7, 25.5, 70.7, 58.3 and 79.9%, respectively (Additional file 7: Table S1 and Additional file 8: Figure S7). Across all patients, Simvastatin, Rosuvastatin and Pravastatin had higher overall probability, and they were more effective in reducing IL-6 than other statins in COPD patients.

Additional file 9: Figure S8 showed that statins therapy significantly reduced IL-8, the SMD (95% CI) was − 0.55 (− 74,-0.35), and with a significant moderate heterogeneity (*I*^*2*^ *= 80.4%*). The heterogeneity significant reduced when using sensitivity analysis and exclude one studies [59] (*I*^*2*^ *= 0.0%*), the SMD (95% CI) was − 0.37 (− 0.58,-0.16). Subgroup analysis showed Atorvastatin, Rosuvastatin and Simvastatin were significantly reduced IL-8, and the SMD (95% CI) were − 0.28 (− 0.57,0.01), − 0.62 (− 1.06,-0.17) and − 0.86 (− 1.18,-0.53), respectively.

**Fig. 6:**
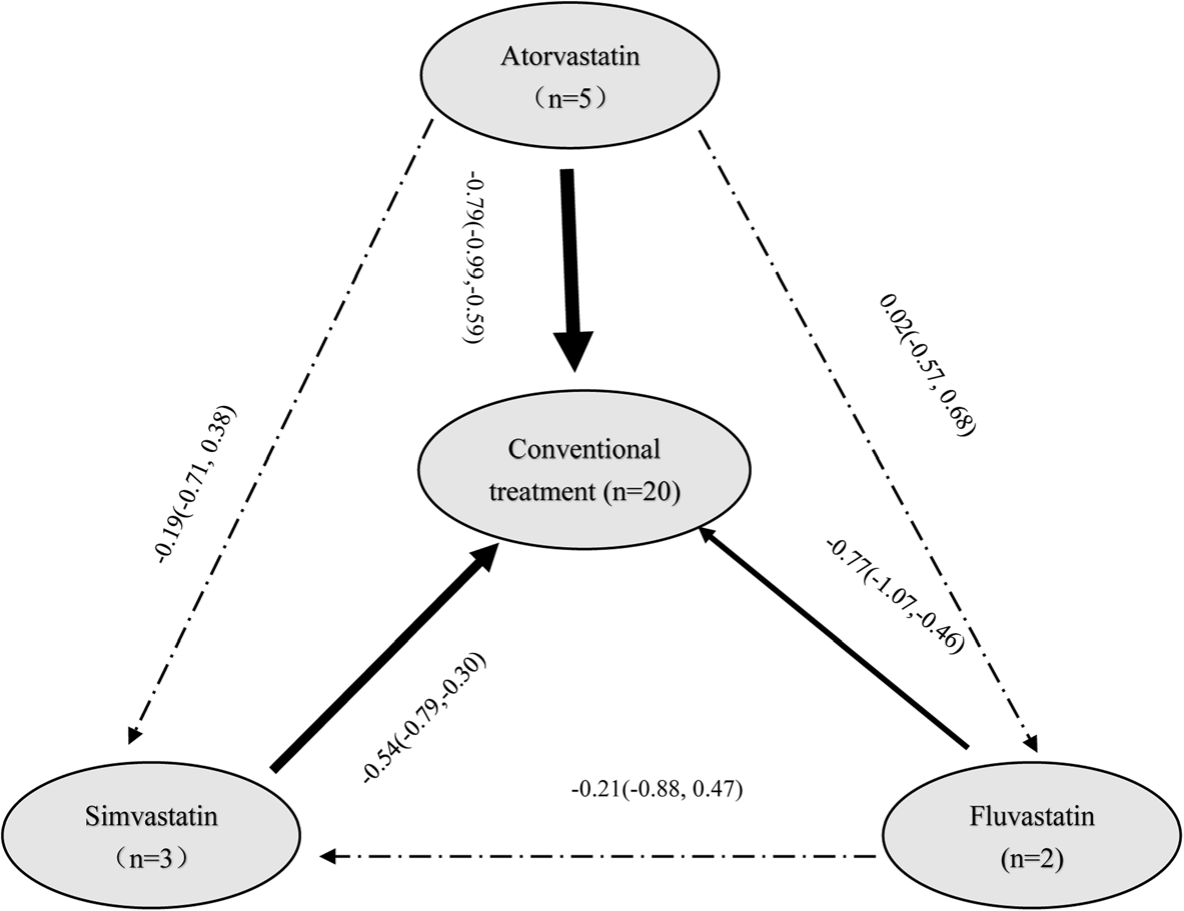
Evidence network of the effect of using statins on PH levels in COPD patients

Additional file 10: Figure S9 showed that statins therapy significantly reduced TNF-α, the SMD (95% CI) was − 0.68 (− 0.83,-0.52), with a significant moderate heterogeneity (*I*^*2*^ *= 82.3%*). The heterogeneity significant reduced when using sensitivity analysis and exclude one study [59] (*I*^*2*^ *= 65.2%*), the SMD (95% CI) was − 0.56 (− 0.72,-0.39). Subgroup analysis showed Simvastatin and Atorvastatin were significantly reduced TNF-α, and the SMD (95% CI) were − 0.79 (− 0.98,-0.60) and − 0.40 (− 0.69,-0.11), respectively. Additional file 11: Figure S10 showed the diagram of network meta-analysis between the changes of TNF-α after using different statins in COPD patients. Network meta-analysis showed that the SUCRA of reducing the TNF-α in COPD patients in Atorvastatin and Simvastatin were 51.0 and 89.2%, respectively (Additional file 12: Table S2 and Additional file 13: Figure S11). Across all patients, Simvastatin had higher overall probability, and it was more effective in reducing TNF-α than Atorvastatin in COPD patients.

**Fig. 7:**
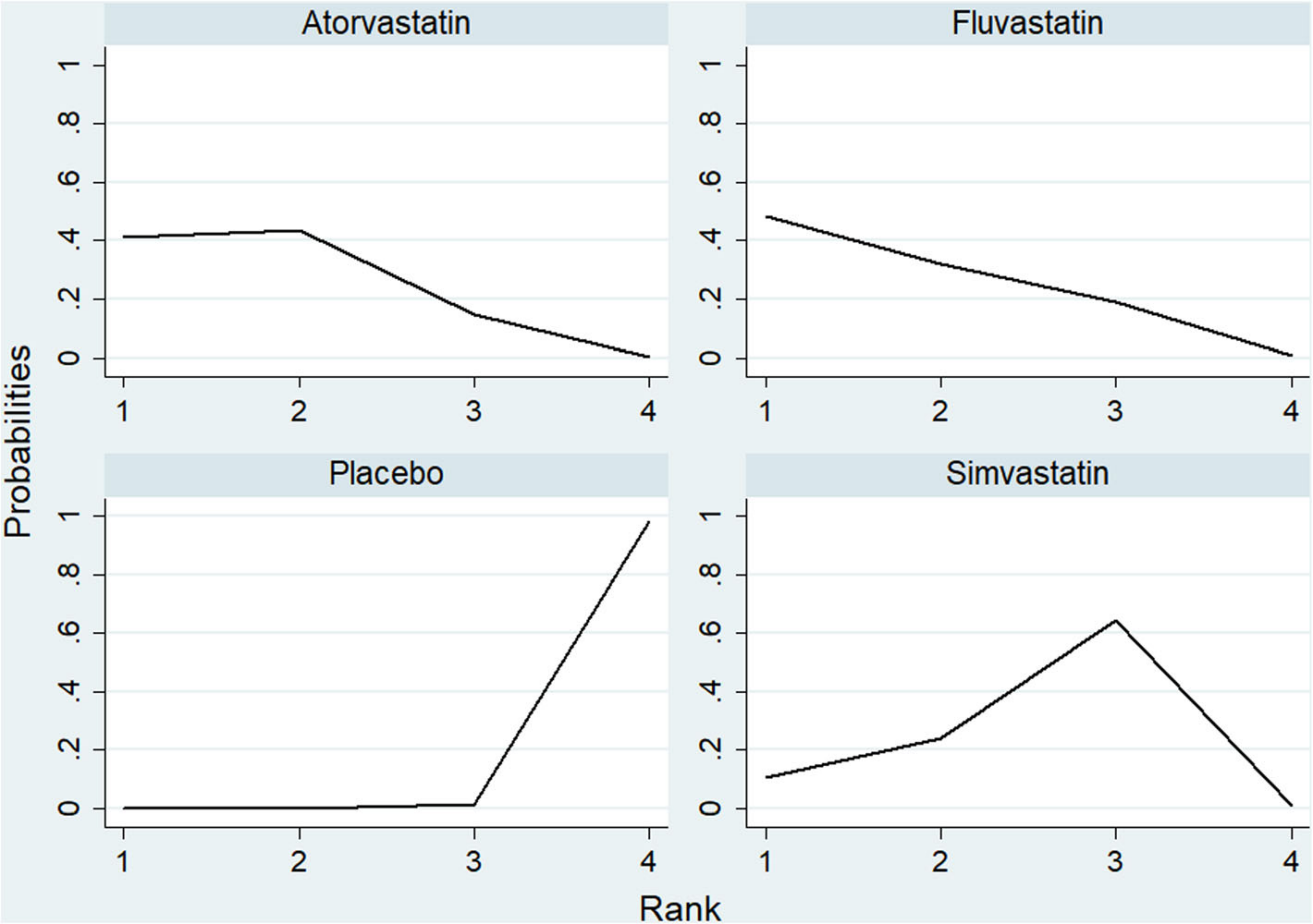
Rank probability analysis of PH with using statins in COPD patients

### Using statins reduced the level of PH

Figure 5 showed that statins significantly reduced PH, the SMD (95% CI) was − 0.71 (− 0.85,-0.57), with a significant lower heterogeneity (*I*^*2*^ *= 45.9%*). Subgroup analysis showed Simvastatin, Atorvastatin and Fluvastatin significantly reduced PH, and the SMD (95% CI) were − 0.54 (− 0.79,-0.30), − 0.79 (− 0.99,-0.59) and − 0.77 (− 1.07,-0.46), respectively. Figure 6 showed the diagram of network meta-analysis between the changes of PH after using different statins in COPD patients. Network meta-analysis showed, the SUCRA of reducing the PH in COPD patients in Atorvastatin, Fluvastatin and Simvastatin were 75.4, 76.0 and 48.0%, respectively (Table 3 and Fig. 7). Across all patients, Fluvastatin and Atorvastatin had higher overall probability, and they were more effective in increasing FEV% than Simvastatin in COPD patients.

Additional file 14: Figure S12 showed that statins significantly increased FEV1%, the SMD (95% CI) was 0.38 (0.28, 0.49), with a significant moderate heterogeneity (*I*^*2*^ *= 66.0%*). The heterogeneity significantly reduced when using sensitivity analysis and exclude one study [74] (*I*^*2*^ *= 36.9%*), the SMD (95% CI) was 0.46 (0.35, 0.57). Subgroup analysis showed Pravastatin, Rosuvastatin, Simvastatin and Atorvastatin were significantly increased FEV1%, and the SMD (95% CI) were 0.29 (0.09, 0.50), 0.72 (0.41, 1.02), 0.41 (0.22,0.59) and 0.64 (0.39,0.90), respectively. Additional file 15: Figure S13 showed the diagram of network meta-analysis between the changes of FEV1% after using different statins in COPD patients. Network meta-analysis showed that Fluvastatin significantly increased compared with Atorvastatin, the SMD (95% CI) was 0.79 (0.03, 1.41). The SUCRA of increasing the FEV1% in COPD patients in Atorvastatin, Fluvastatin, Rosuvastatin, Pravastatin and Simvastatin were 17.0, 91.2, 11.0, 54.2 and 42.3%, respectively (Additional file 16: Table S3 and Additional file 17: Figure S14). Across all patients, Fluvastatin, Pravastatin and Simvastatin had higher overall probability, and they were more effective in increasing FEV1% than other statins in COPD patients.

Additional file 18: Figure S15 showed that statins significantly increased FEV1/FVC%, the SMD (95% CI) was 0.35 (0.23, 0.47), with a significant strong heterogeneity (*I*^*2*^ *= 81.3%*). The heterogeneity significantly reduced when using sensitivity analysis and exclude one study [74] (*I*^*2*^ *= 48.2%*), the SMD (95% CI) was 0.49 (0.08, 0.64). Subgroup analysis showed that Rosuvastatin, Simvastatin, Atorvastatin and Pravastatin were significantly increased FEV1/FVC%, and the SMD (95% CI) were 0.41 (0.11, 0.71), 0.53 (0.34,0.72), 0.63 (0.33,0.92) and 0.36 (0.08,0.64), respectively. Additional file 19: Figure S16 showed the diagram of network meta-analysis between the changes of FEV1/FVC% after using different statins in COPD patients. Network meta-analysis showed, the SUCRA of increasing the FEV1/FVC% in COPD patients in Atorvastatin, Fluvastatin, Rosuvastatin, Pravastatin and Simvastatin were 19.4, 93.3, 36.2, 45.3 and 30.3%, respectively (Additional file 20: Table S4 and Additional file 21: Figure S17). Across all patients, Fluvastatin, Pravastatin and Rosuvastatin had higher overall probability, and they were more effective in increasing FEV1/FVC% than other statins in COPD patients.

Additional file 22: Figure S18 showed that statins significantly increased 6 MW, the SMD (95% CI) was 0.54 (0.38, 0.71), with a significant lower heterogeneity (*I*^*2*^ *= 40.8%*). Subgroup analysis showed Atorvastatin and Simvastatin were significantly increased 6WMD, and the SMD (95% CI) were 0.42 (0.14, 0.70) and 0.79 (0.52,1.05), respectively. Additional file 23: Figure S19 showed the diagram of network meta-analysis between the changes of 6 MW after using different statins in COPD patients. Network meta-analysis showed, the SUCRA of increasing the 6 MW in COPD patients in Atorvastatin, Rosuvastatin, Pravastatin and Simvastatin were 30.5, 46.6, 43.1 and 47.8%, respectively (Additional file 24: Table S5 and Additional file 25: Figure S20). Across all patients, Simvastatin and Rosuvastatin had higher overall probability, and they were more effective in increasing 6 MW than other statins in COPD patients.

### Using statins reduced the level of TC and TG

Additional file 26: Figure S21 showed that statins significantly reducing TC, the SMD (95% CI) was − 0.86 (− 1.02, − 0.70), with a significant moderate heterogeneity (*I*^*2*^ *= 71.1%*). The heterogeneity significantly reduced when using sensitivity analysis and exclude one study [74] (*I*^*2*^ *= 53.7%*), the SMD (95% CI) was − 0.75 (− 0.92,-0.57). Subgroup analysis showed that Pravastatin, Simvastatin, Atorvastatin an Fluvastatin significantly reduced TC, and the SMD (95% CI) were − 1.02 (− 1.34,-0.70), − 0.82 (− 1.07,-0.57), − 0.69 (− 1.30,-0.09) and − 1.58 (− 2.00,-1.15), respectively. Additional file 27: Figure S22 showed the diagram of network meta-analysis between the changes of TC after using different statins in COPD patients. Network meta-analysis showed that Fluvastatin significantly reduced TC compared Simvastatin, the SMD (95% CI) was − 0.77 (− 1.47,-0.07). The SUCRA of reducing the TC in COPD patients in Atorvastatin, Fluvastatin, Rosuvastatin, Pravastatin and Simvastatin were 39.5, 96.9, 51.1, 65.1 and 46.4%, respectively (Additional file 28: Table S6 and Additional file 29: Figure S23). Across all patients, Fluvastatin, Pravastatin and Rosuvastatin had higher overall probability, and they were more effective in reducing TC than other statins in COPD patients.

Additional file 30: Figure S24 showed that statins significantly reduced TG, the SMD (95% CI) was − 0.21 (− 0.39,-0.02), with a significant stronger heterogeneity (*I*^*2*^ *= 87.7%*). The heterogeneity significantly reduced when using sensitivity analysis and exclude one study [74] (*I*^*2*^ *= 60.2%*), the SMD (95% CI) was − 0.50 (− 0.71,-0.29). Subgroup analysis showed Pravastatin and Atorvastatin were significantly reducing TG. The SMD (95% CI) were − 0.66 (− 0.97,-0.35) and − 1.01 (− 1.55, − 0.47), respectively. Additional file 31: Figure S25 showed the diagram of network meta-analysis between the changes of TG using different statins in COPD patients. Network meta-analysis showed, Atorvastatin significantly reduced TG compared Fluvastatin, the SMD (95% CI) was − 1.75 (− 3.13, − 0.28). The SUCRA of reducing the TG in COPD patients in Atorvastatin, Fluvastatin, Rosuvastatin, Pravastatin and Simvastatin were 91.6, 5.3, 48.5, 78.7 and 39.4%, respectively (Additional file 32: Table S7 and Additional file 33: Figure S26). Across all patients, Atorvastatin, Pravastatin and Rosuvastatin had higher overall probability, and they were more effective in reducing TG than other statins in COPD patients.

### Sensitivity analysis and publication bias

We saw heterogeneity among studies about the *I*^*2*^ of all-cause mortality, heart disease-related mortality, COPD mortality and AECOPD were 86.8, 60.7, 95.7 and 78.4%. The heterogeneity were significantly reduced after removing studies with a large heterogenous source through sensitivity analysis. The heterogeneity of inflammatory factors, lung function index, blood lipids index significantly reduced through removing studies with a large heterogenous source by sensitivity analysis or subgroups analysis. Begger regression tests provided no evidence of substantial publication bias (*P > 0.05* for all tests).

## Discussion

Our study used general meta-analysis to estimate the relative risk of morality in using statins, and used network meta-analysis to estimate standardized mean difference between different statins, and to further determine their effectiveness in COPD in reducing inflammatory factors and increasing lung function index, and the most important is to find which statins have more effective for treating COPD patients. Our network meta-analysis not only included the long-term outcomes which involving all-cause and cause-specific mortality, but also included the recent outcomes which containing inflammatory factors, lung function index, blood lipids index and PH, which comprehensively evaluated which statin is better for COPD patients and provide evidence for the clinical treatment.

The study found that using statins reduced 29% of the risk of all-cause mortality, 28% COPD mortality and 16% AECOPD, the RR (95% CI) were 0.71 (0.62, 0.80), 0.72 (0.53, 0.98) and 0.84 (0.79, 0.89), respectively. A recent meta-analysis found [16] using statins reduced all-cause mortality, COPD mortality and AECOPD which including twenty studies published 2017 years, the HR (95% CI) were 0.65 (0.57–0.74), 0.41 (0.28–0.59) and 0.58 (0.48–0.72), respectively. However, it didn’t study COPD death. Zhang et al. found [17] that statins therapy was associated with reduced pH which including six studies, the SMD (95% CI) was − 0.70 (− 0.99, − 0.41). PH is a serious and progressive vascular disease, which is yet incurable and generally results in heart failure and death. Similarly, our study also found using statins reduced PH which including ten studies, the SMD (95% CI) was − 0.71 (− 0.85,-0.57). Subgroup analysis found that Simvastatin, Atorvastatin and Fluvastatin significantly reduced PH, and the SMD (95% CI) were − 0.54 (− 0.79,-0.30), − 0.79 (− 0.99,-0.59) and − 0.77 (− 1.07,-0.46), respectively.

Network meta-analysis showed the SUCRA of Atorvastatin, Fluvastatin and Simvastatin were 75.4, 76.0 and 48.0%, which means Atorvastatin and Fluvastatin are more effective than simvastatin. The well-known mechanisms for the occurrence and development of PH include pulmonary vascular remodeling, abnormal vasoconstriction, and thrombosis, which all lead to increased pulmonary arterial pressure and vascular resistance, as well as right heart failure. Seeming to aim at these pathophysiological alterations, statins have been shown to restrict vascular cell proliferation, improve endothelial function by increasing Nitric oxide (NO) production, and inhibiting thrombogenic response, thus may exert a potential therapeutic benefit [76, 77].

Chronic inflammation and oxidation reaction played a huge role in the progression of COPD [78]. The protective effect of statins could be explained by their pleiotropic effects, including inhibit vascular endothelial inflammatory response, stabilize athermanous plaque, antithrombotic effects, and improve endothelial function [79]. As COPD progresses, lots of inflammatory biomarkers, such as CRP, IL-6, IL-8, and TNF-α increased in COPD patients [80]. These numerous inflammatory responses are believed to contribute to disease progression of COPD, and systemic inflammation often together with COPD and elevated circulating inflammatory factors can be an important risk factor for mortality [81]. Our study found using statin reduced inflammatory mediators including CRP, IL-6, IL-8 and TNF-α, the SMD (95% CI) were − 0.62 (− 0.52,-0.72), − 0.95 (− 1.09,-0.81), − 0.37 (− 0.58,-0.16) and − 0.56 (− 0.72,-0.39), respectively. Subgroup analysis showed that there were different effectiveness using different statins in reducing inflammatory mediators. Network meta-analysis showed Fluvastatin (97.7%), Atorvastatin and Rosuvastatin had higher overall probability, and they were more effective in reducing CRP than other statins in COPD patients; Simvastatin, Rosuvastatin and Pravastatin had higher overall probability, and they were more effective in reducing IL-6 than other statins in COPD patients. A large number of studies have showed the effect of statins to modulate the immune responses and reduce inflammatory mediators including CRP, IL-6, IL-8, and IL-17 in inflammatory based diseases [82, 83].

Wright et al. study showed that endothelin (ET) is a powerful pulmonary vasodilator and plays an important role in the pathogenesis and treatment of COPD patients [84]. NO has been known as maintain patency. The interaction between ET and NO together maintains the tone of the blood vessels. When vascular endothelial cells are damaged, endothelin is synthesized and released in large amounts, but it does not increase the synthesis and release of NO, resulting in an imbalance of ET and NO, and the formation of pulmonary artery remodeling. Studies have shown that fluvastatin can increase the post-transcriptional activity of the eNOS gene, inhibition of endothelin synthesis and secretion, promotion of NO synthesis and secretion, and improve lung function [56, 85]. Chronic inflammatory response can also stimulate vascular endothelial cells and smooth muscle cells to produce ET, promote the formation of PH. Atorvastatin can reduce the secretion of IL-6 and TNF-ɑ, reduce the release of CRP, reduce the inflammatory response, regulate the balance of NO and ET-1, and ultimately reduce the pulmonary artery pressure and Improve lung function by inhibiting the transcription of nuclear factor (NF)-KB [73, 82]. A common complication of COPD is PH, which mechanism is complex and not fully understood. Study has found that chronic hypoxia induced PH, the main mechanism may be associated with hypoxia-induced pulmonary vasoconstriction, which in turn leads to dysfunction of pulmonary vascular endothelial cells, promotes increased synthesis and clearance of ET-1, and inhibits NO synthesis and release, the pulmonary blood vessels gradually remodel and eventually develop into PH [86]. Soo speculates that CRP and ET-1 may be closely related to PH in patients with COPD [87]. Based on the results of this study, we speculated that statins can be used to prevent COPD progression and reduce PH by reducing CRP, IL-6 and TNF-ɑ and other inflammatory factors.

The present study has a number of advantages over the previous meta-analysis. On the one hand, this study not only includes long-term outcome indicators such as the risk of AECOPD, heart disease-related mortality and all-cause mortality, but also includes inflammatory factors and lung function index, comprehensive analysis the effect of using statin in COPD patients. On the other hand, network meta-analysis studied which statin is better for treating COPD. Of course, there are some limitations of the study. Firstly, in the study of the risk of AECOPD, heart disease-related mortality and all-cause mortality, the original studies did not refer to specific statins, so only general meta-analysis was performed. Secondly, although our study included a comparison of the effects of different statins, the results which indirect were less effective than the direct comparisons. Thirdly, RCTs included in the study have a small sample size. Therefore, there is needed more and more randomized, well-designed, multi-center, direct comparison and double blind clinical studies to identify the long-term effects of statins in COPD patients.

In conclusion, our network meta-analysis showed that long-term using statins reduced inflammatory factors including CRP and IL-6, increased lung function index including FEV1% and FEV1/FVC%, and reduced the risk of AECOPD, heart disease-related mortality and all-cause mortality. In addition, Fluvastatin, Atorvastatin and Rosuvastatin are more effective in COPD patients considered comprehensively.

## Conclusion

Using statins can reduce the risk of mortality, the level of CRP and PH in COPD patients. In addition, Fluvastatin and Atorvastatin are more effective in reducing CRP and PH in COPD patients.

## Additional files

Additional file 1: Flow chart of studies search and studies selection. (TIF 12473 kb)

Additional file 2: Forest plot showing effect of statins on heart disease-related mortality in COPD patients. (TIF 39534 kb)

Additional file 3: Forest plot showing effect of statins on COPD mortality in COPD patients. (TIF 39315 kb)

Additional file 4: Forest plot showing effect of statins on AECOPD in COPD patients. (TIF 36283 kb)

Additional file 5: Forest plot showing effect of statins on IL-6 in COPD patients. (TIF 29566 kb)

Additional file 6: Evidence network of the effect of using statins on IL-6 levels in COPD patients. (TIF 14099 kb)

Additional file 7: Rank probability analysis of IL-6 with using statins in COPD patients. (PDF 177 kb)

Additional file 8: Rank probability analysis of IL-6 with using statins in COPD patients. (TIF 36014 kb)

Additional file 9: Forest plot showing effect of statins on IL-8 in COPD patients. (TIF 34035 kb)

Additional file 10: Forest plot showing effect of statins on TNF-α in COPD patients. (TIF 34092 kb)

Additional file 11: Evidence network of the effect of using statins on TNF-α levels in COPD patients. (TIF 8093 kb)

Additional file 12: Rank probability analysis of TNF-α with using statins in COPD patients. (PDF 272 kb)

Additional file 13: Rank probability analysis of TNF-α with using statins in COPD patients. (TIF 36012 kb)

Additional file 14: Forest plot showing effect of statins on FEV1% in COPD patients. (TIF 28073 kb)

Additional file 15: Evidence network of the effect of using statins on FEV1% in COPD patients. (TIF 13811 kb)

Additional file 16: Rank probability analysis of FEV1% with using statins in COPD patients. (PDF 177 kb)

Additional file 17: Rank probability analysis of FEV1% with using statins in COPD patients. (TIF 36014 kb)

Additional file 18: Forest plot showing effect of statins on FEV1/FVC% in COPD patients. (TIF 28661 kb)

Additional file 19: Evidence network of the effect of using statins on FEV1/FVC% in COPD patients. (TIF 13672 kb)

Additional file 20: Rank probability analysis of FEV1/FVC% with using statins in COPD patients. (PDF 177 kb)

Additional file 21: Rank probability analysis of FEV1/FVC% with using statins in COPD patients. (TIF 36014 kb)

Additional file 22: Forest plot showing effect of statins on 6MW in COPD patients. (TIF 32244 kb)

Additional file 23: Evidence network of the effect of using statins on 6MW in COPD patients. (TIF 13184 kb)

Additional file 24: Rank probability analysis of 6MW with using statins in COPD patients. (PDF 176 kb)

Additional file 25: Rank probability analysis of 6MW with using statins in COPD patients. (TIF 36013 kb)

Additional file 26: Forest plot showing effect of statins on TC in COPD patients. (TIF 31508 kb)

Additional file 27: Evidence network of the effect of using statins on TC levels in COPD patients. (TIF 13642 kb)

Additional file 28: Rank probability analysis of TC with using statins in COPD patients. (PDF 177 kb)

Additional file 29: Rank probability analysis of TC with using statins in COPD patients. (TIF 36014 kb)

Additional file 30: Forest plot showing effect of statins on TG in COPD patients. (TIF 32559 kb)

Additional file 31: Evidence network of the effect of using statins on TG levels in COPD patients. (TIF 13633 kb)

Additional file 32: Rank probability analysis of TG with using statins in COPD patients. (PDF 177 kb)

Additional file 33: Rank probability analysis of TG with using statins in COPD patients. (TIF 36014 kb)
