# Supplementary figures and images for: Effectiveness of long-term using statins in COPD – a network meta-analysis
Source: Respir Res. 2019 Jan 23;20:17. doi: 10.1186/s12931-019-0984-3 (PMC6343315; doi:10.1186/s12931-019-0984-3)

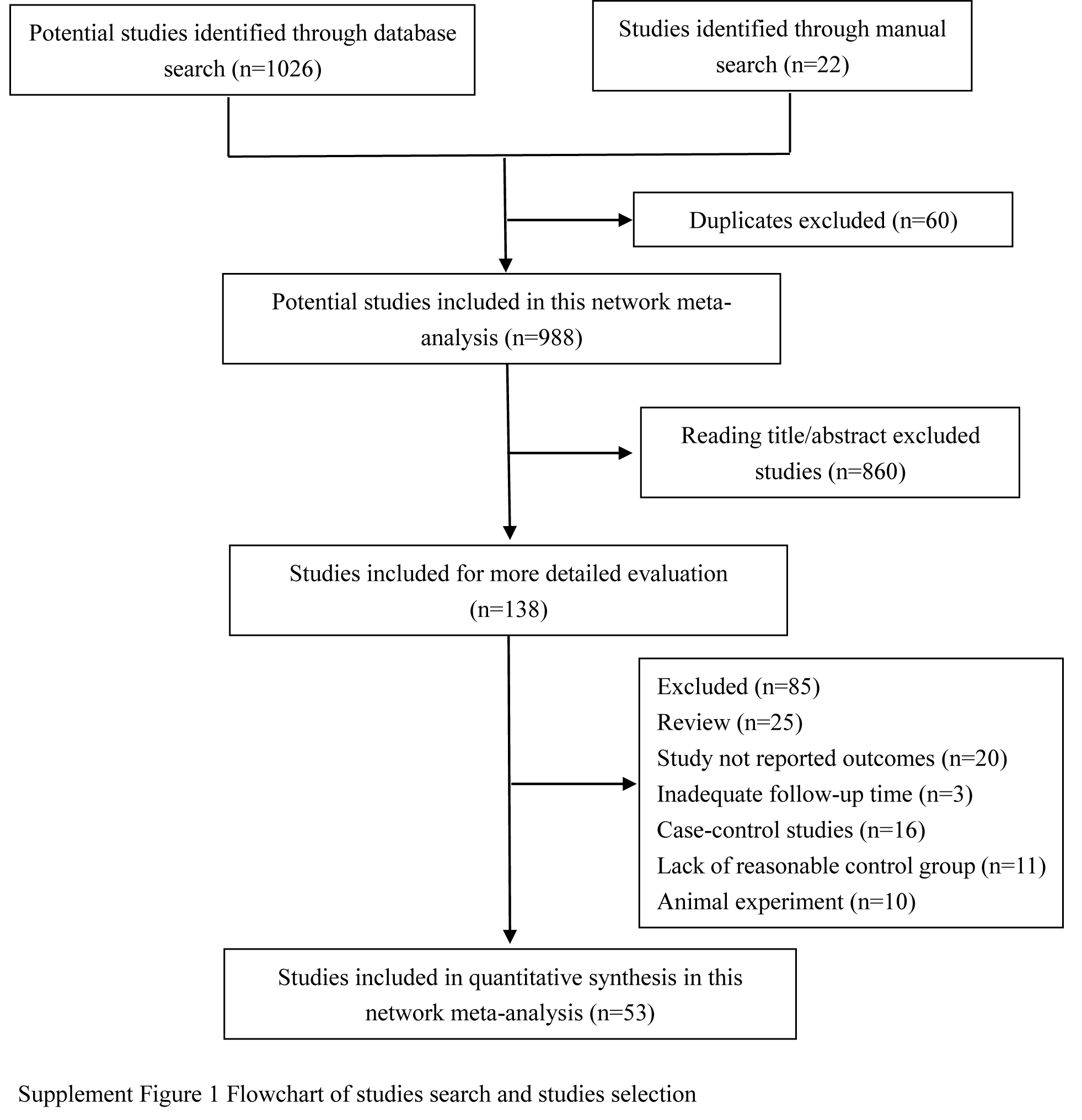

Supplement: Supplementary file 1 — Flow chart of studies search and studies selection. (TIF 12473 kb) [file 12931_2019_984_MOESM1_ESM.tif]

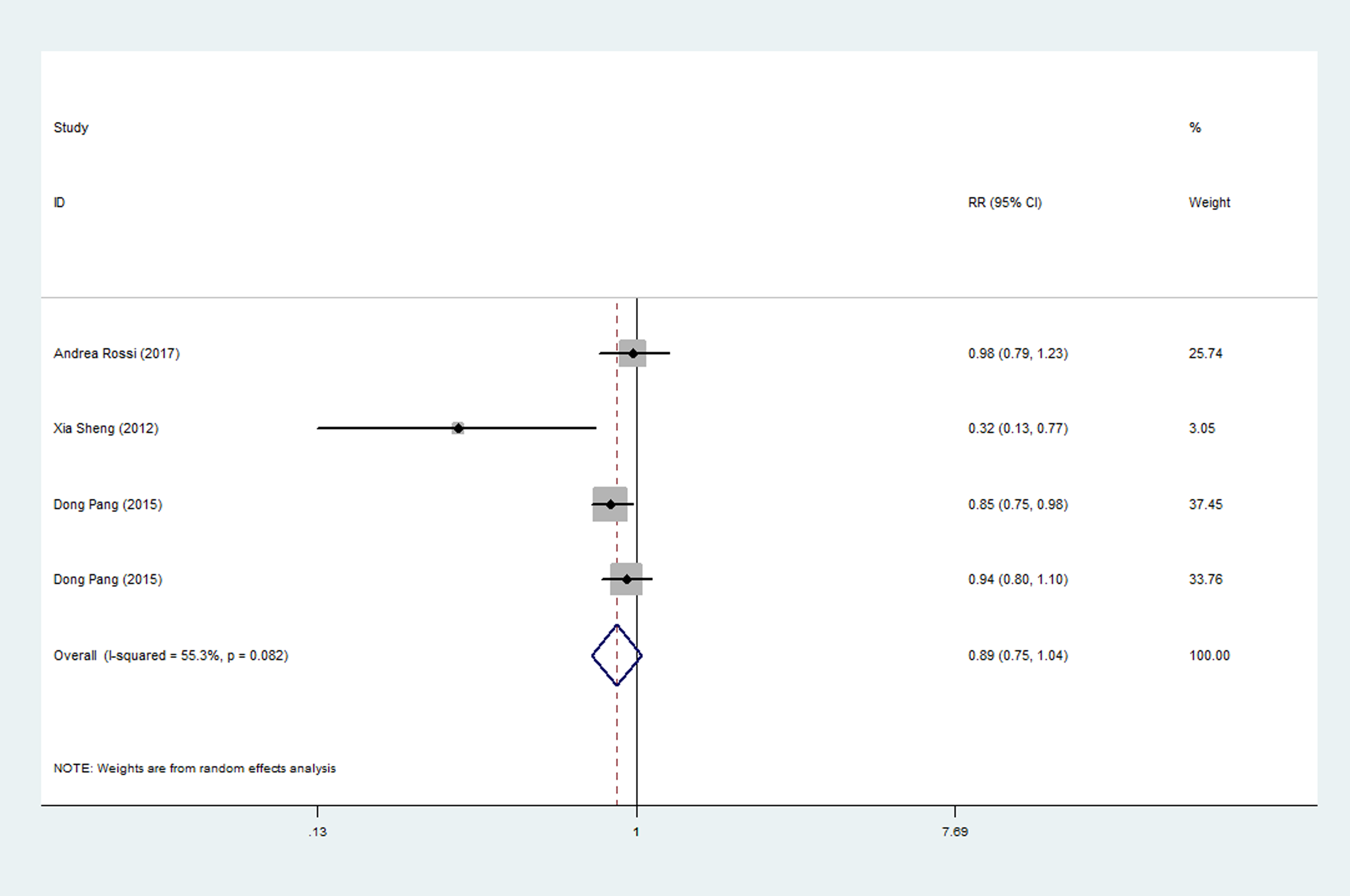

Supplement: Supplementary file 2 — Forest plot showing effect of statins on heart disease-related mortality in COPD patients. (TIF 39534 kb) [file 12931_2019_984_MOESM2_ESM.tif]

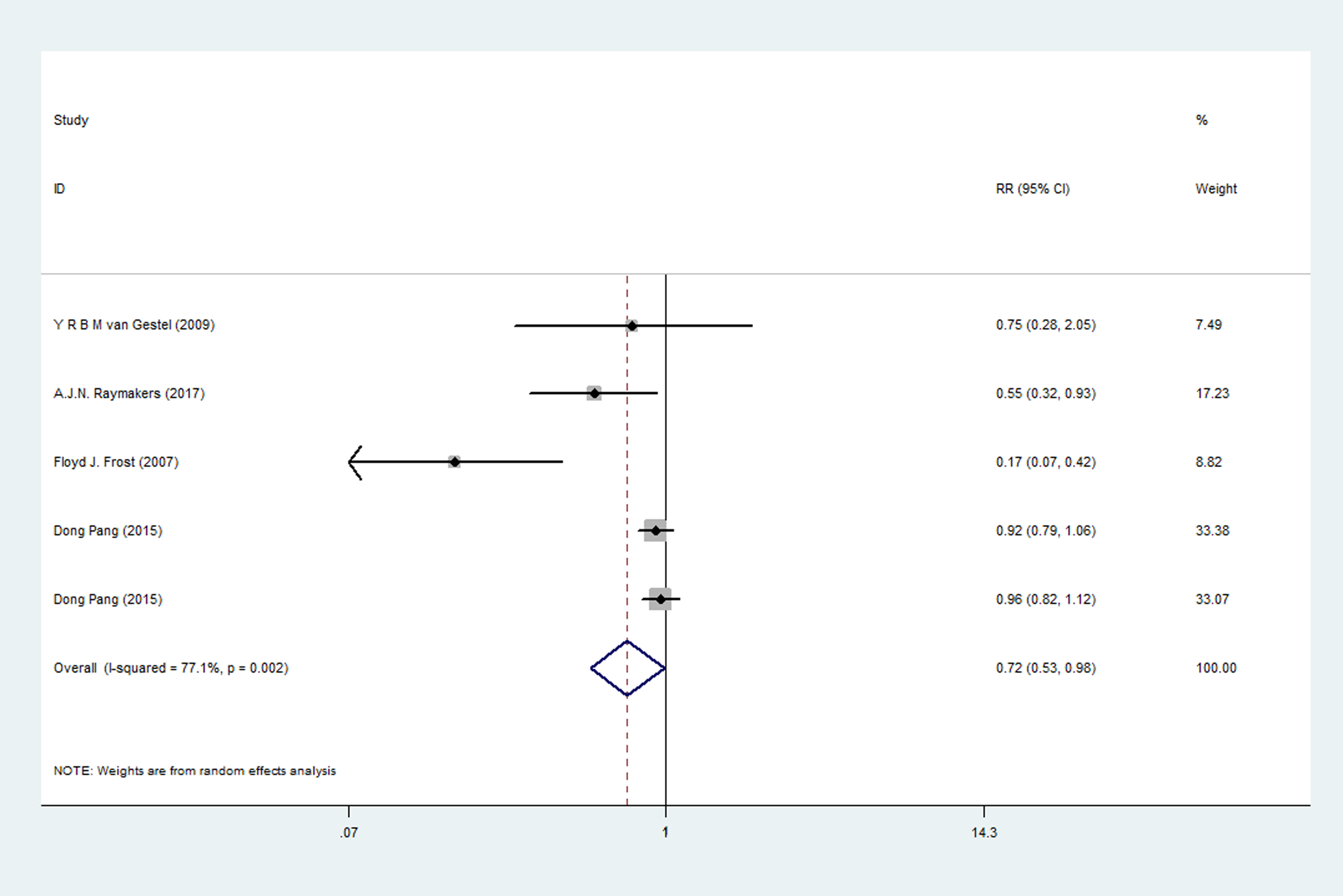

Supplement: Supplementary file 3 — Forest plot showing effect of statins on COPD mortality in COPD patients. (TIF 39315 kb) [file 12931_2019_984_MOESM3_ESM.tif]

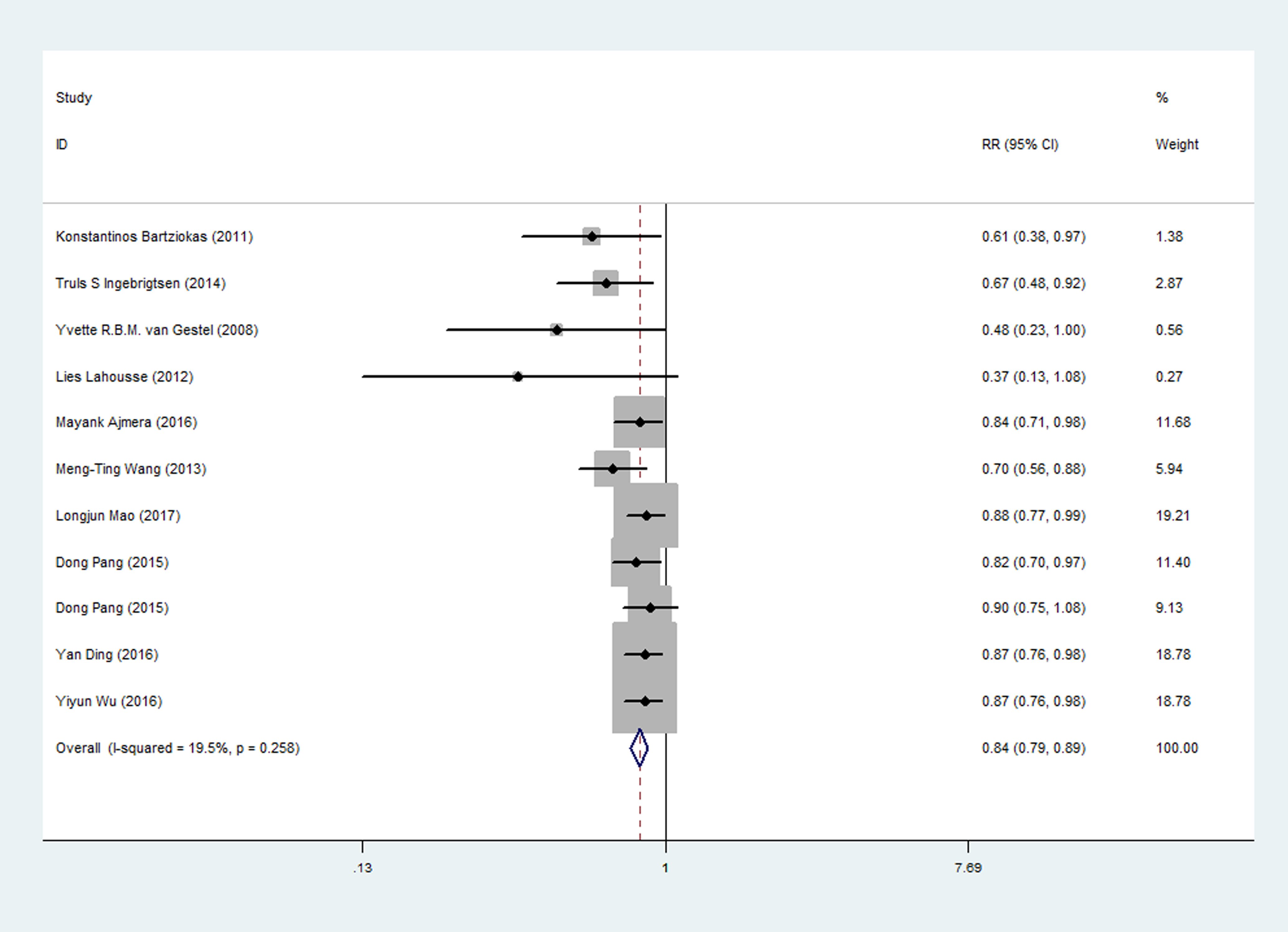

Supplement: Supplementary file 4 — Forest plot showing effect of statins on AECOPD in COPD patients. (TIF 36283 kb) [file 12931_2019_984_MOESM4_ESM.tif]

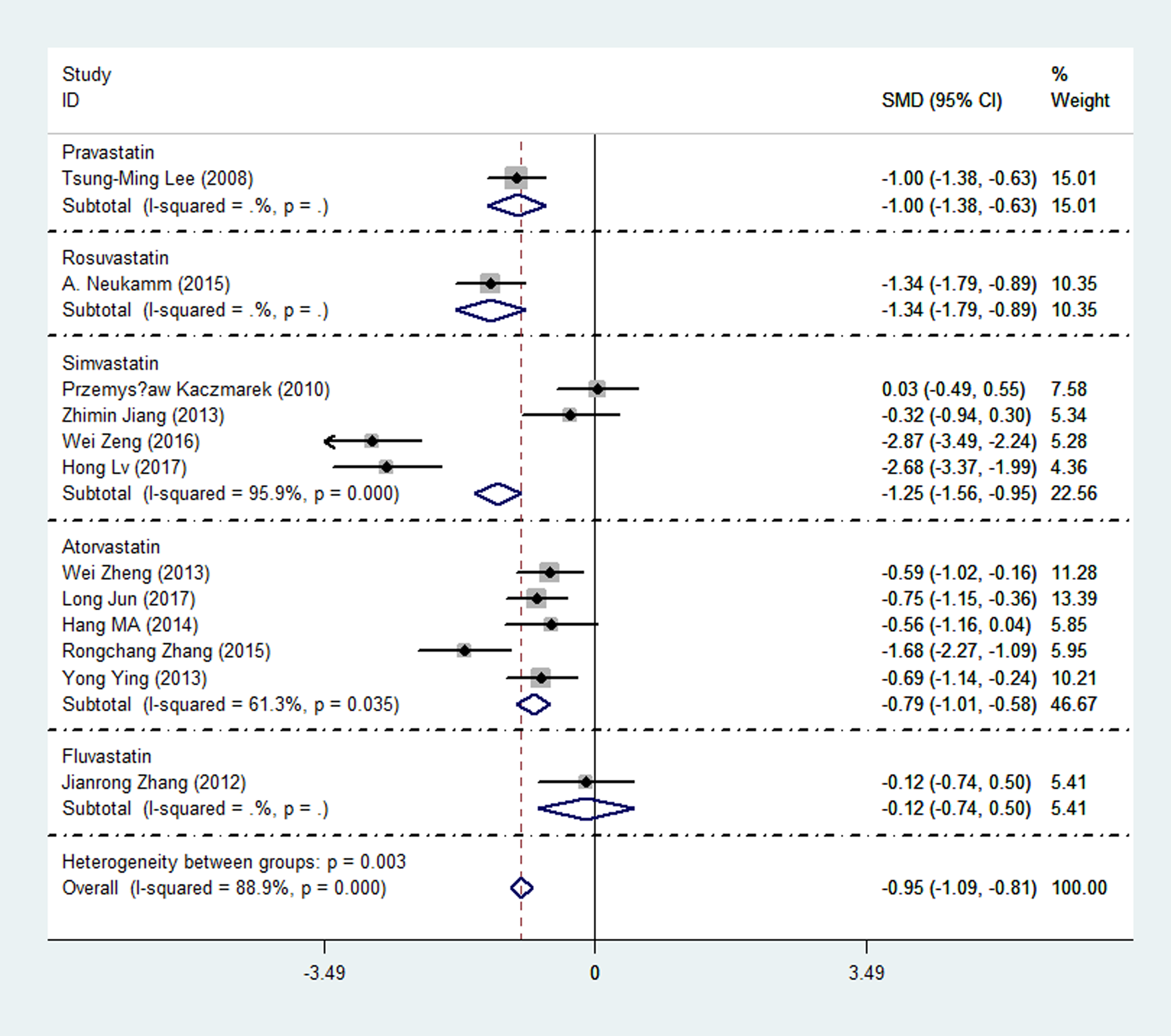

Supplement: Supplementary file 5 — Forest plot showing effect of statins on IL-6 in COPD patients. (TIF 29566 kb) [file 12931_2019_984_MOESM5_ESM.tif]

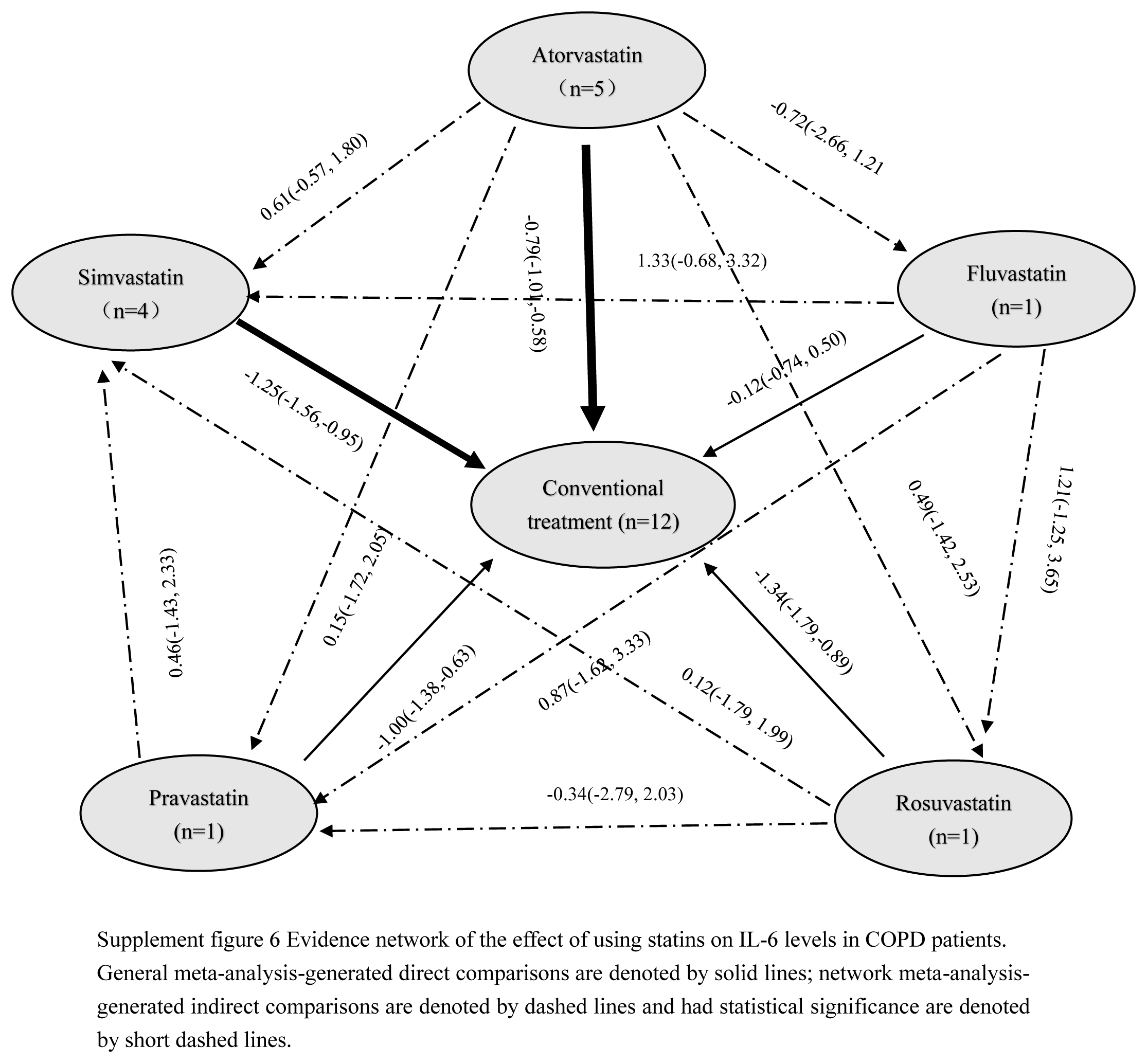

Supplement: Supplementary file 6 — Evidence network of the effect of using statins on IL-6 levels in COPD patients. (TIF 14099 kb) [file 12931_2019_984_MOESM6_ESM.tif]

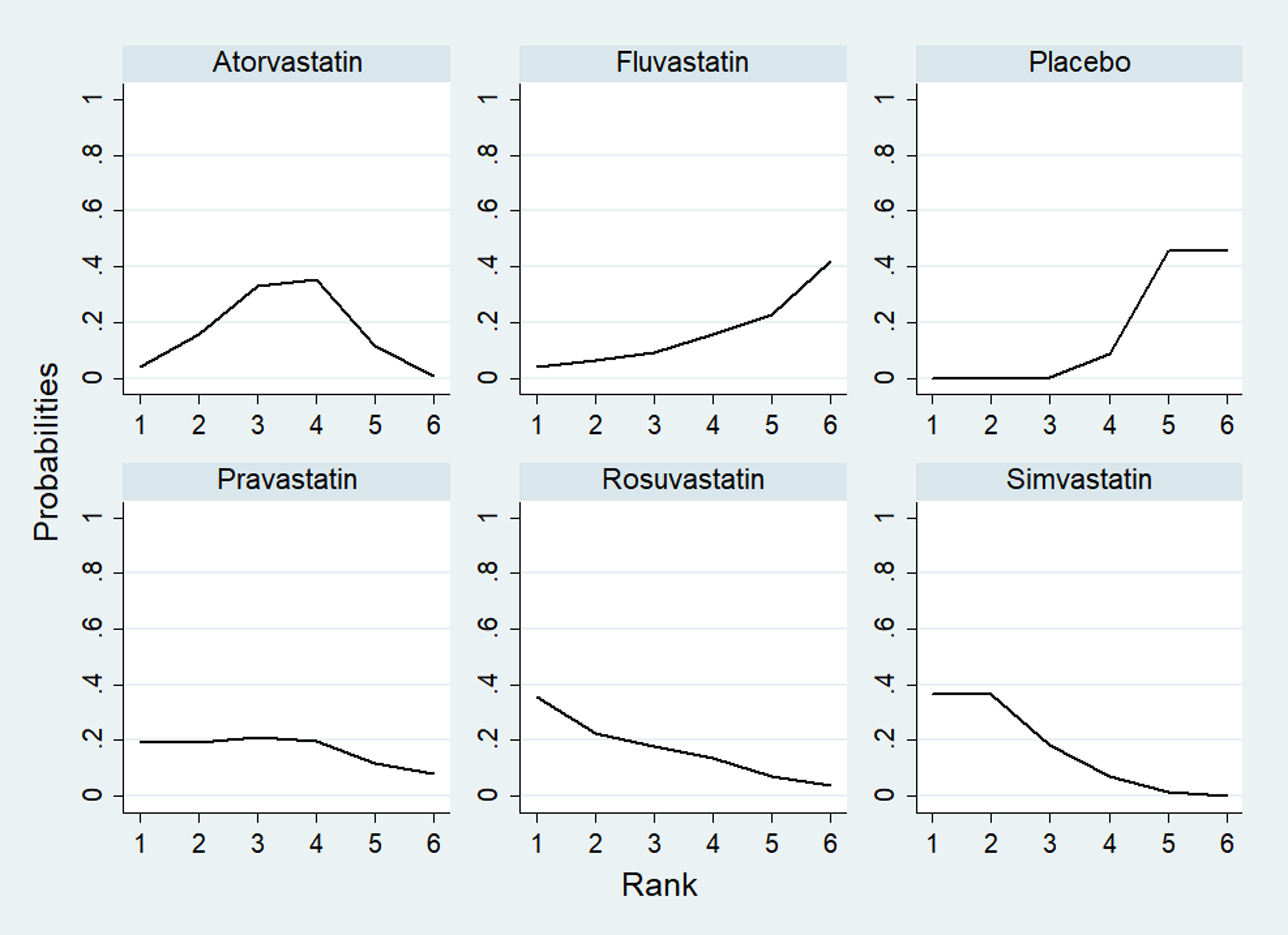

Supplement: Supplementary file 8 — Rank probability analysis of IL-6 with using statins in COPD patients. (TIF 36014 kb) [file 12931_2019_984_MOESM8_ESM.tif]

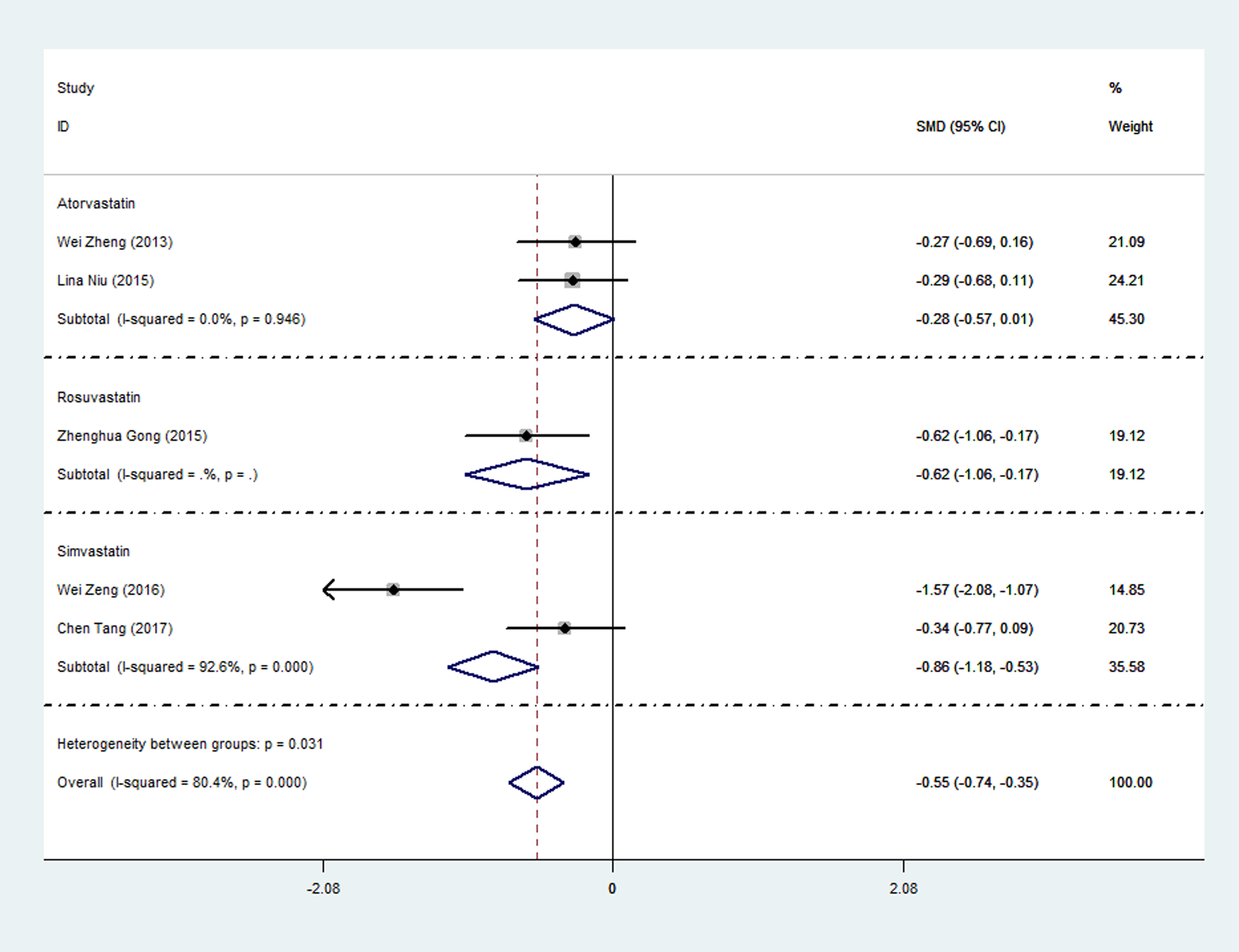

Supplement: Supplementary file 9 — Forest plot showing effect of statins on IL-8 in COPD patients. (TIF 34035 kb) [file 12931_2019_984_MOESM9_ESM.tif]

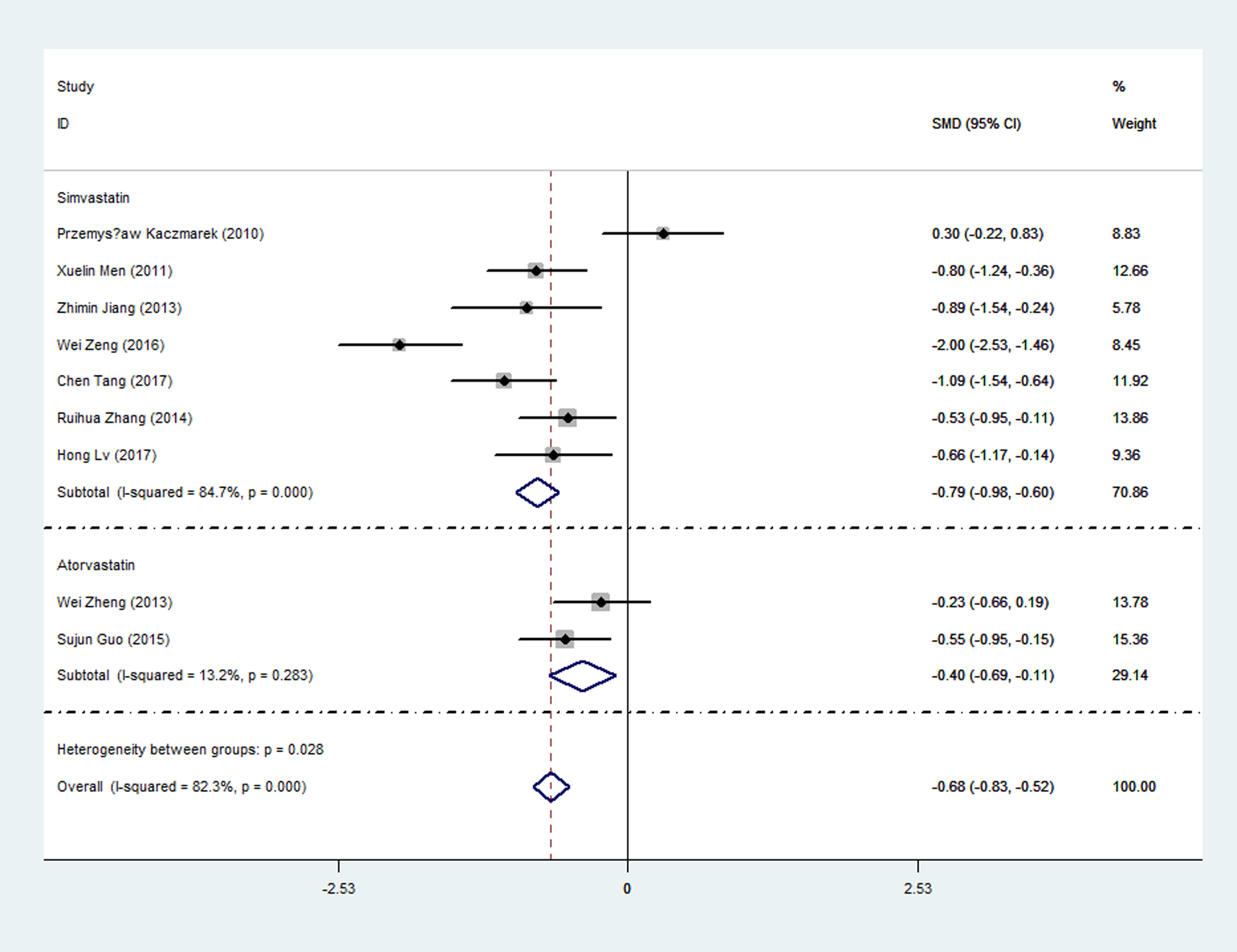

Supplement: Supplementary file 10 — Forest plot showing effect of statins on TNF-α in COPD patients. (TIF 34092 kb) [file 12931_2019_984_MOESM10_ESM.tif]

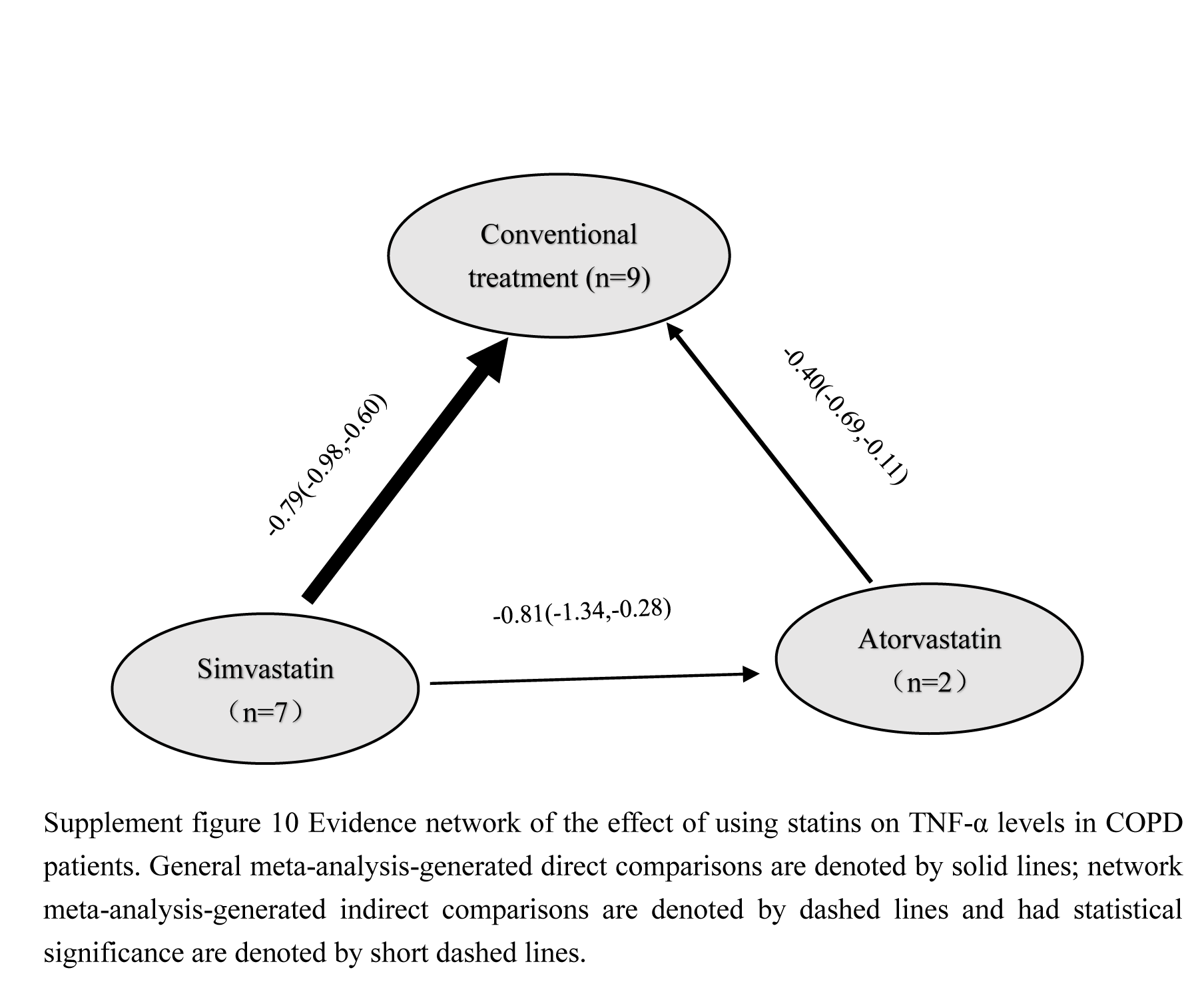

Supplement: Supplementary file 11 — Evidence network of the effect of using statins on TNF-α levels in COPD patients. (TIF 8093 kb) [file 12931_2019_984_MOESM11_ESM.tif]

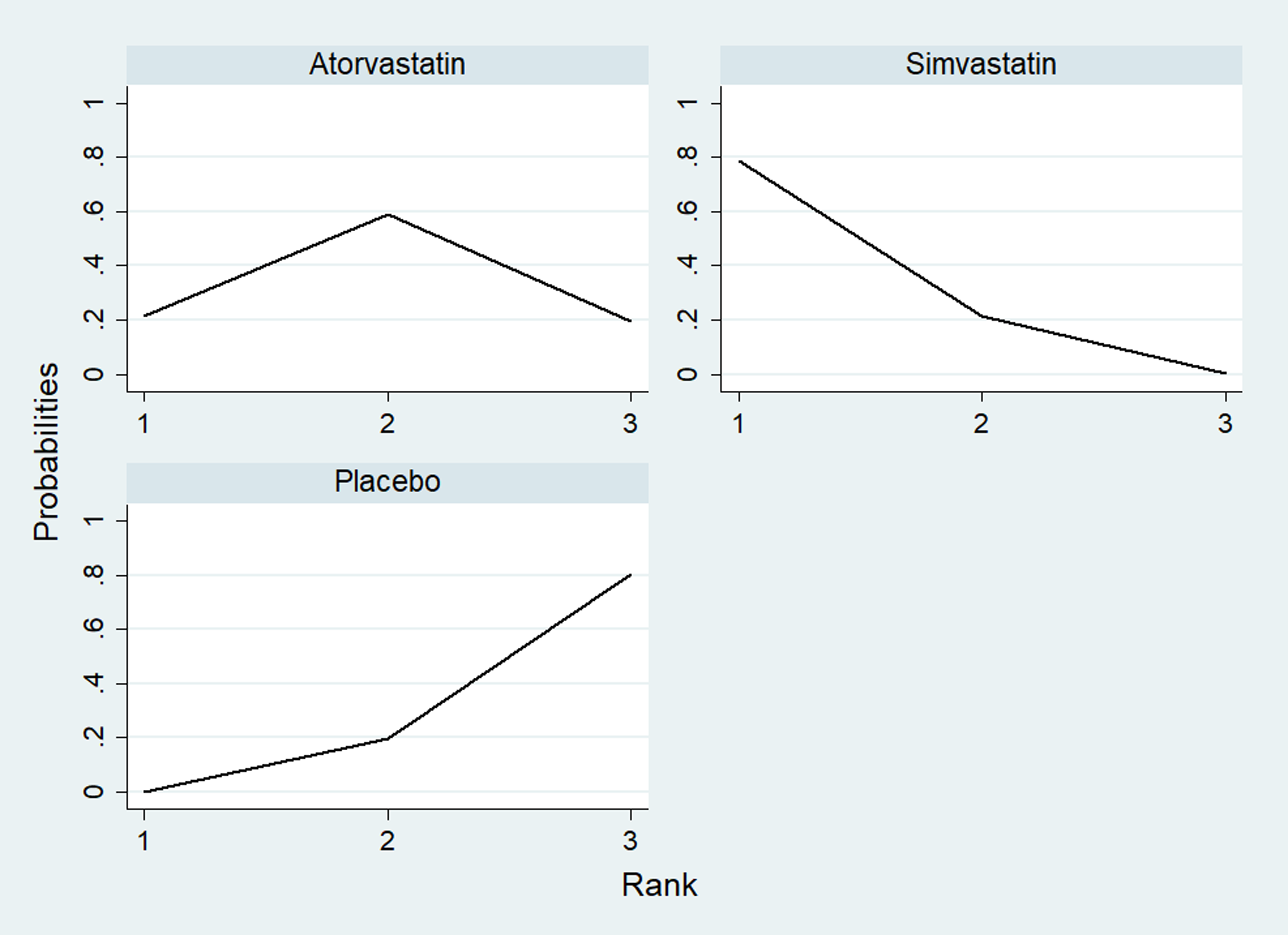

Supplement: Supplementary file 13 — Rank probability analysis of TNF-α with using statins in COPD patients. (TIF 36012 kb) [file 12931_2019_984_MOESM13_ESM.tif]

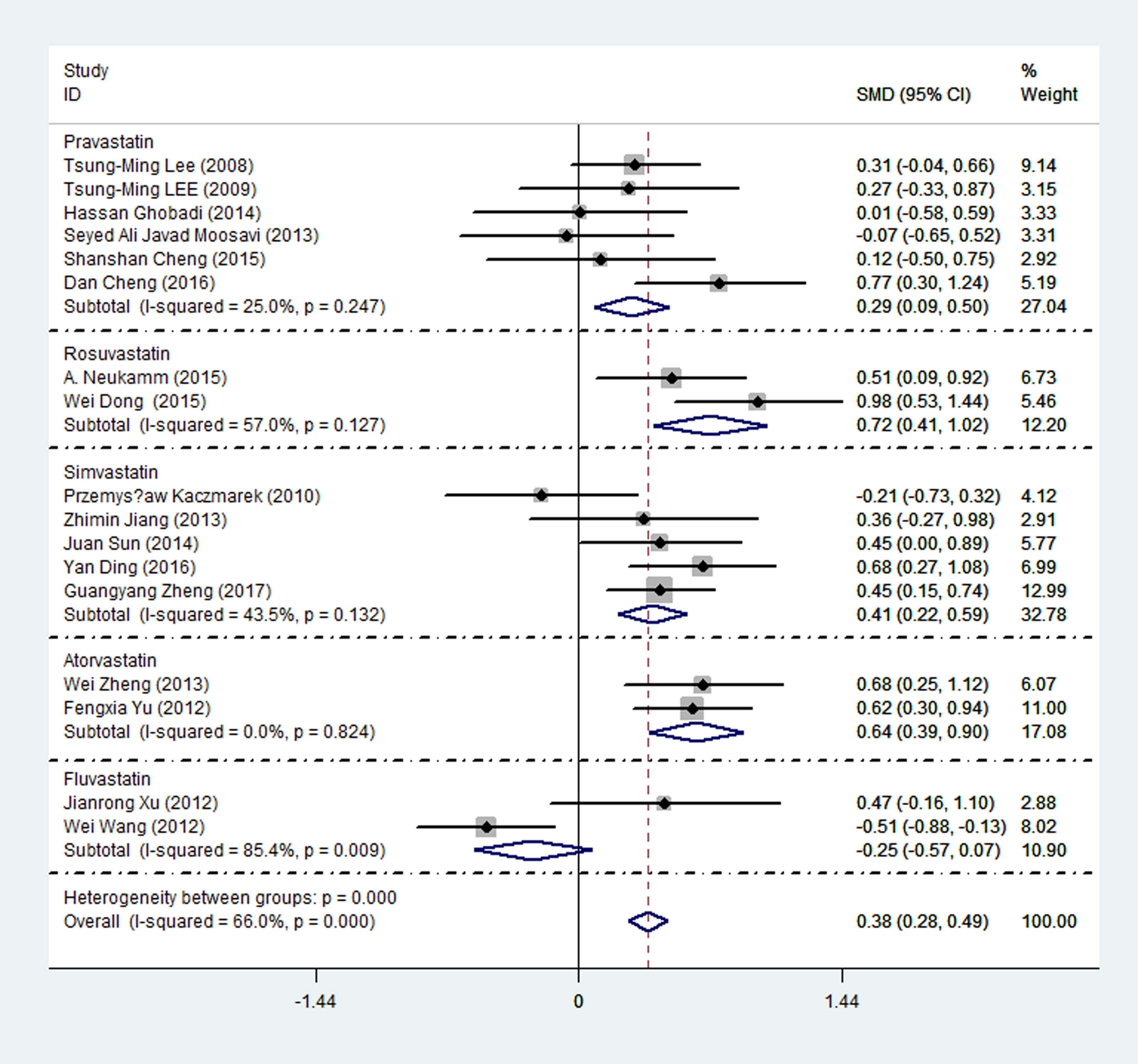

Supplement: Supplementary file 14 — Forest plot showing effect of statins on FEV1% in COPD patients. (TIF 28073 kb) [file 12931_2019_984_MOESM14_ESM.tif]

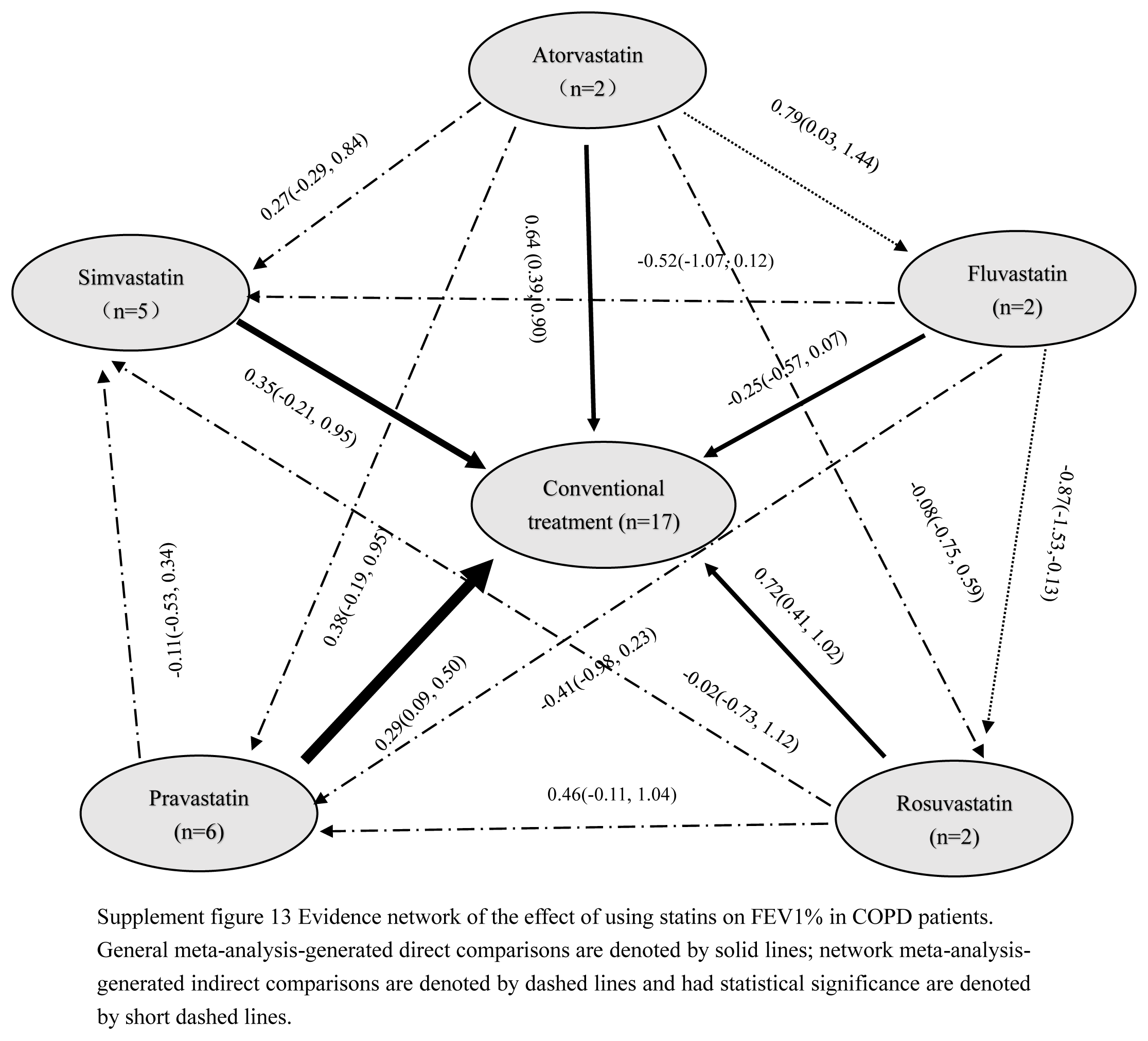

Supplement: Supplementary file 15 — Evidence network of the effect of using statins on FEV1% in COPD patients. (TIF 13811 kb) [file 12931_2019_984_MOESM15_ESM.tif]

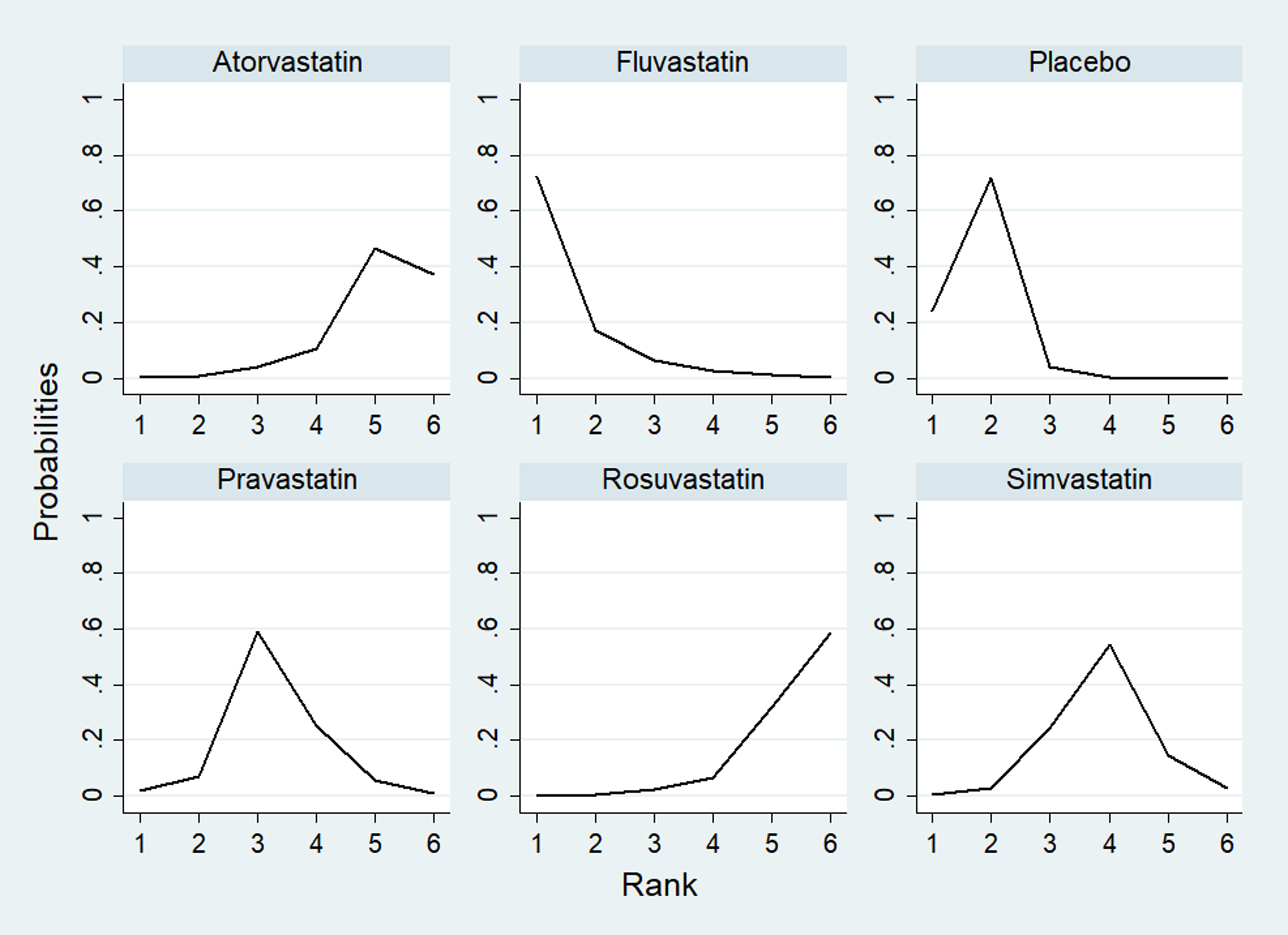

Supplement: Supplementary file 17 — Rank probability analysis of FEV1% with using statins in COPD patients. (TIF 36014 kb) [file 12931_2019_984_MOESM17_ESM.tif]

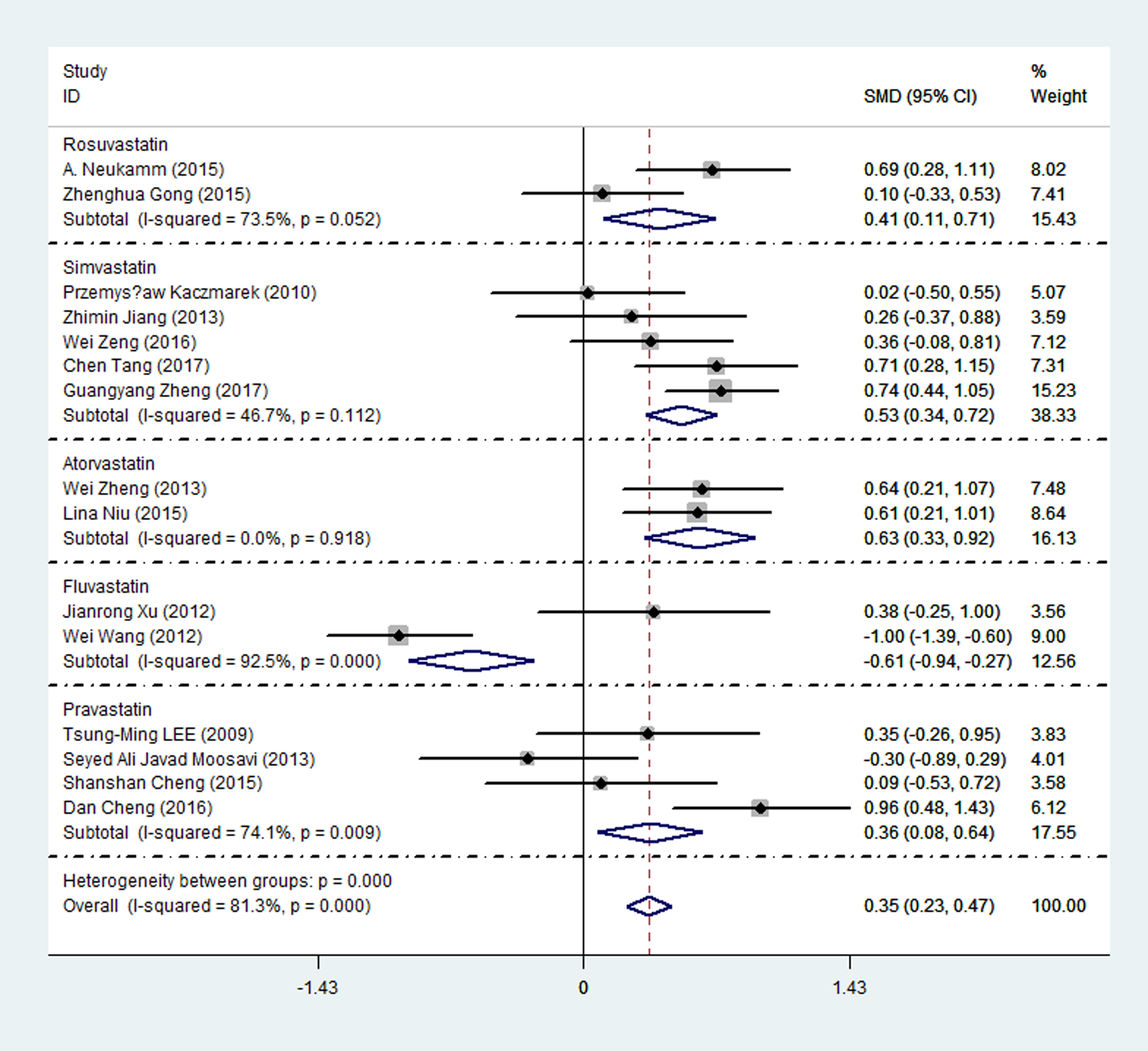

Supplement: Supplementary file 18 — Forest plot showing effect of statins on FEV1/FVC% in COPD patients. (TIF 28661 kb) [file 12931_2019_984_MOESM18_ESM.tif]

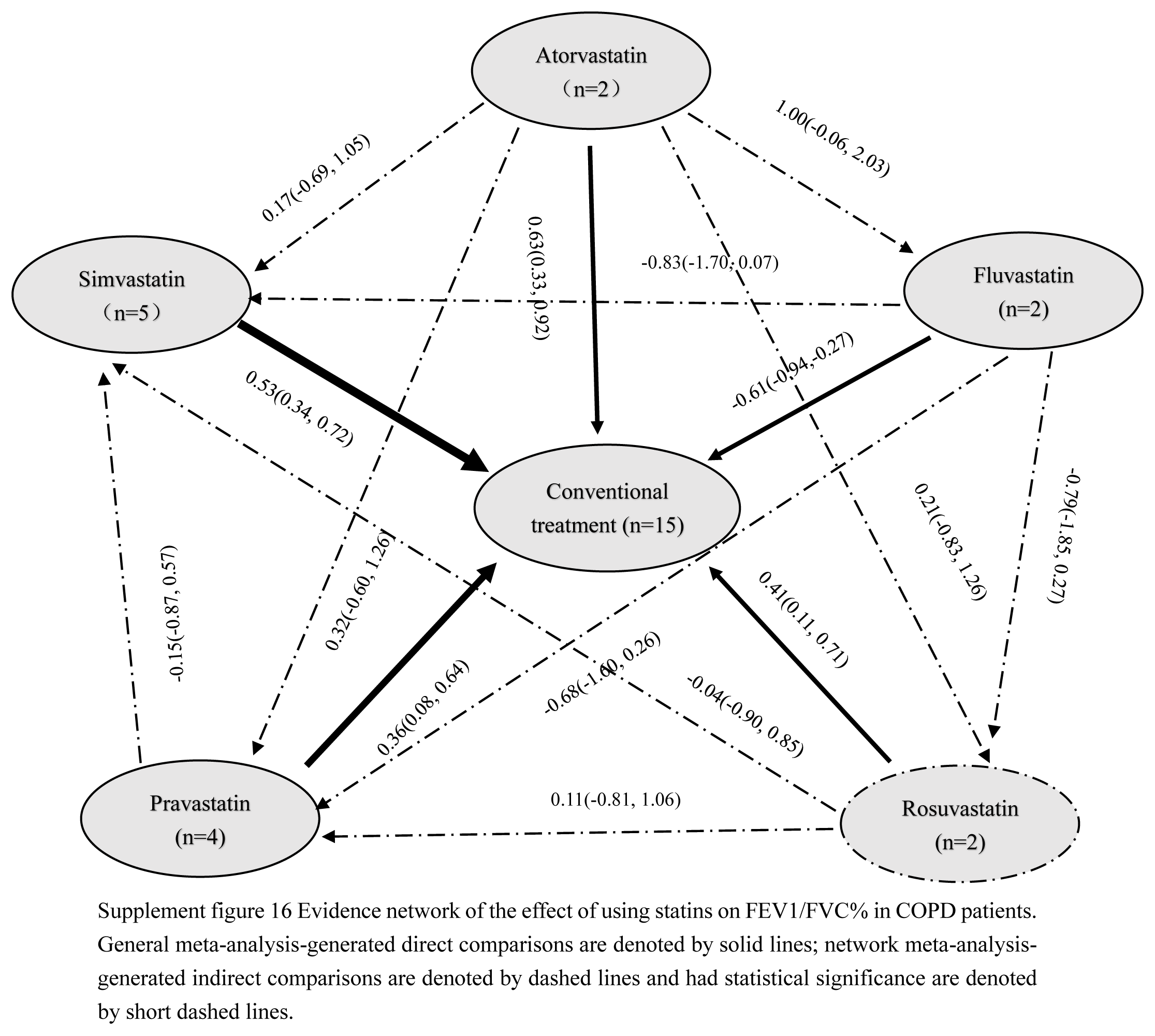

Supplement: Supplementary file 19 — Evidence network of the effect of using statins on FEV1/FVC% in COPD patients. (TIF 13672 kb) [file 12931_2019_984_MOESM19_ESM.tif]

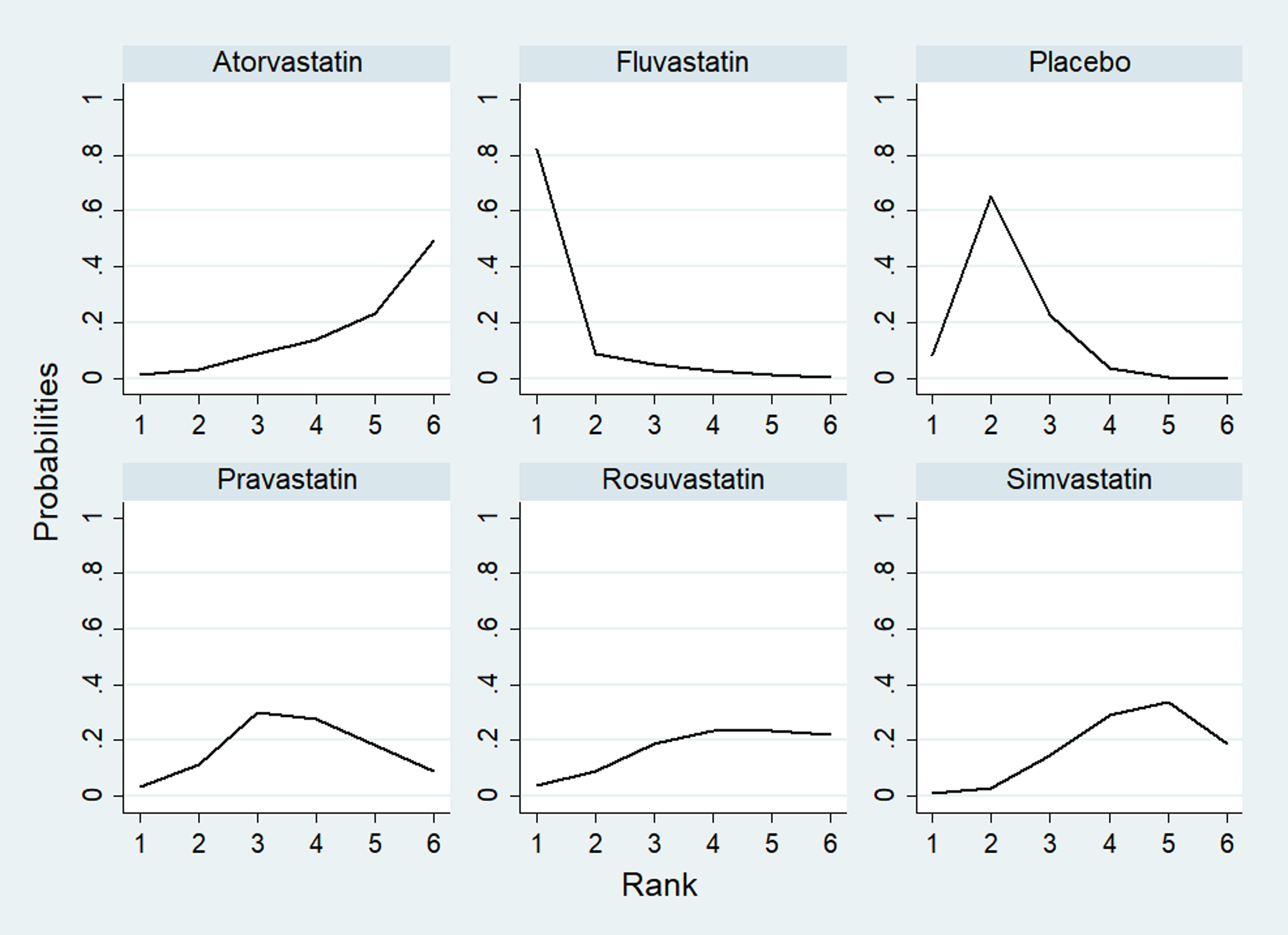

Supplement: Supplementary file 21 — Rank probability analysis of FEV1/FVC% with using statins in COPD patients. (TIF 36014 kb) [file 12931_2019_984_MOESM21_ESM.tif]

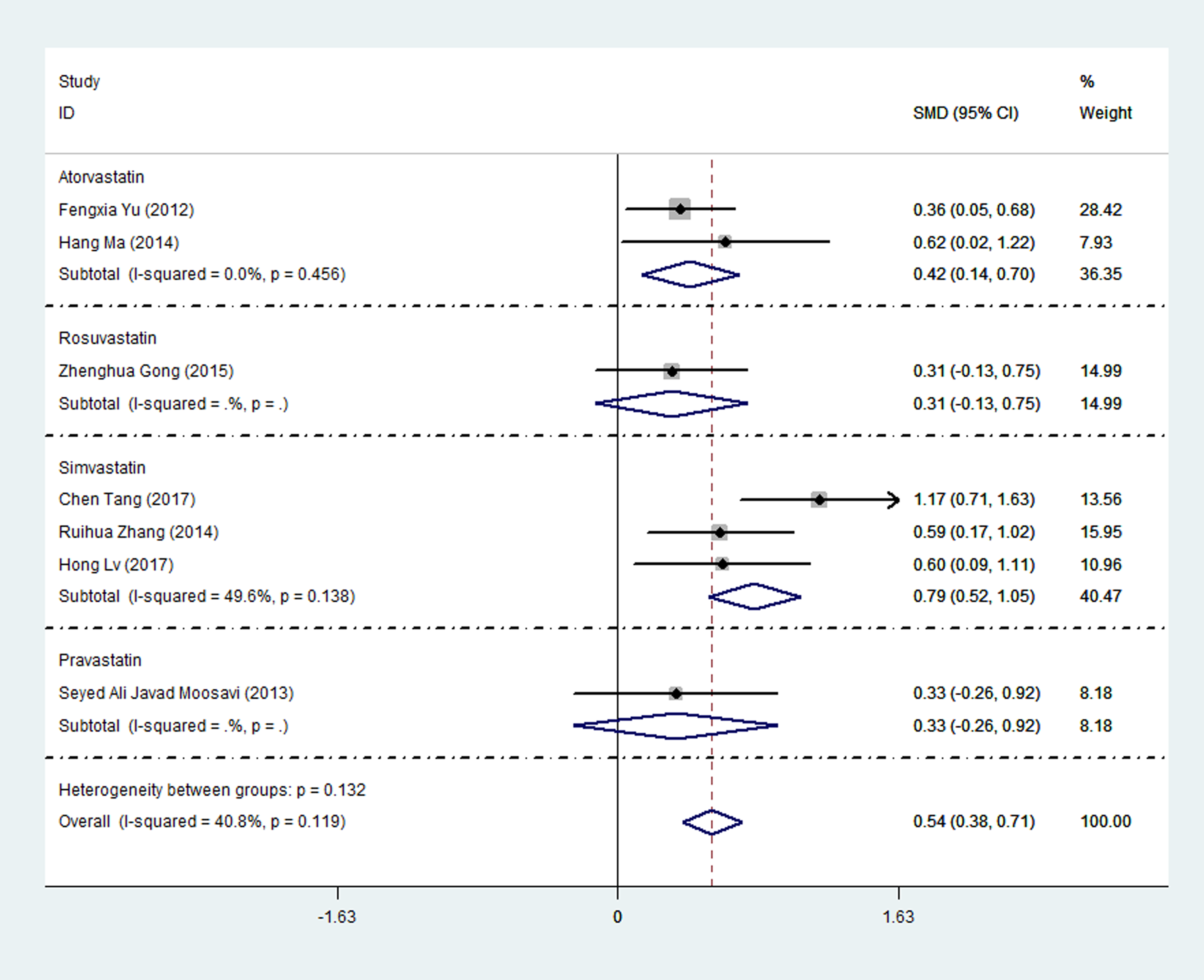

Supplement: Supplementary file 22 — Forest plot showing effect of statins on 6MW in COPD patients. (TIF 32244 kb) [file 12931_2019_984_MOESM22_ESM.tif]

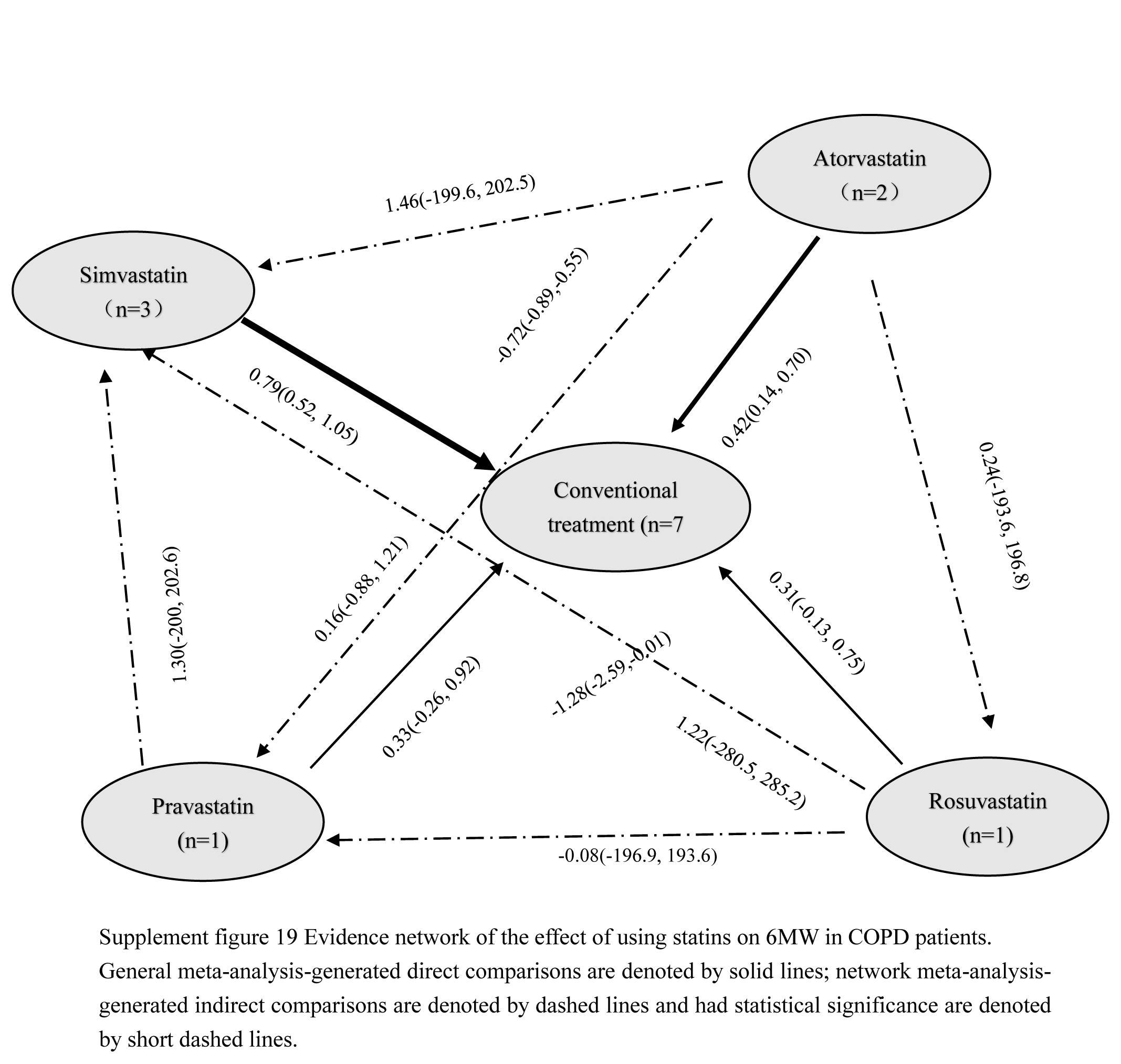

Supplement: Supplementary file 23 — Evidence network of the effect of using statins on 6MW in COPD patients. (TIF 13184 kb) [file 12931_2019_984_MOESM23_ESM.tif]

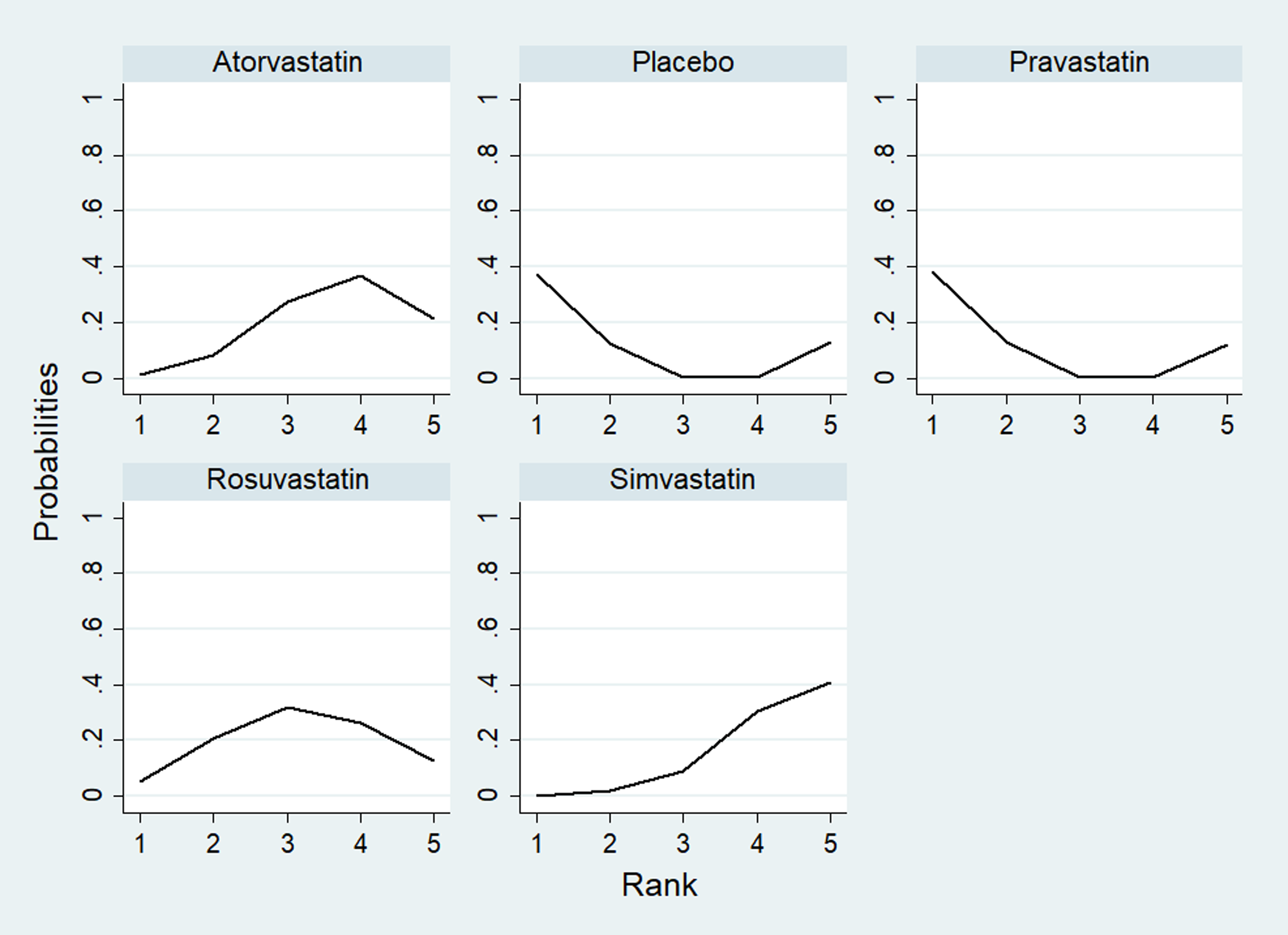

Supplement: Supplementary file 25 — Rank probability analysis of 6MW with using statins in COPD patients. (TIF 36013 kb) [file 12931_2019_984_MOESM25_ESM.tif]

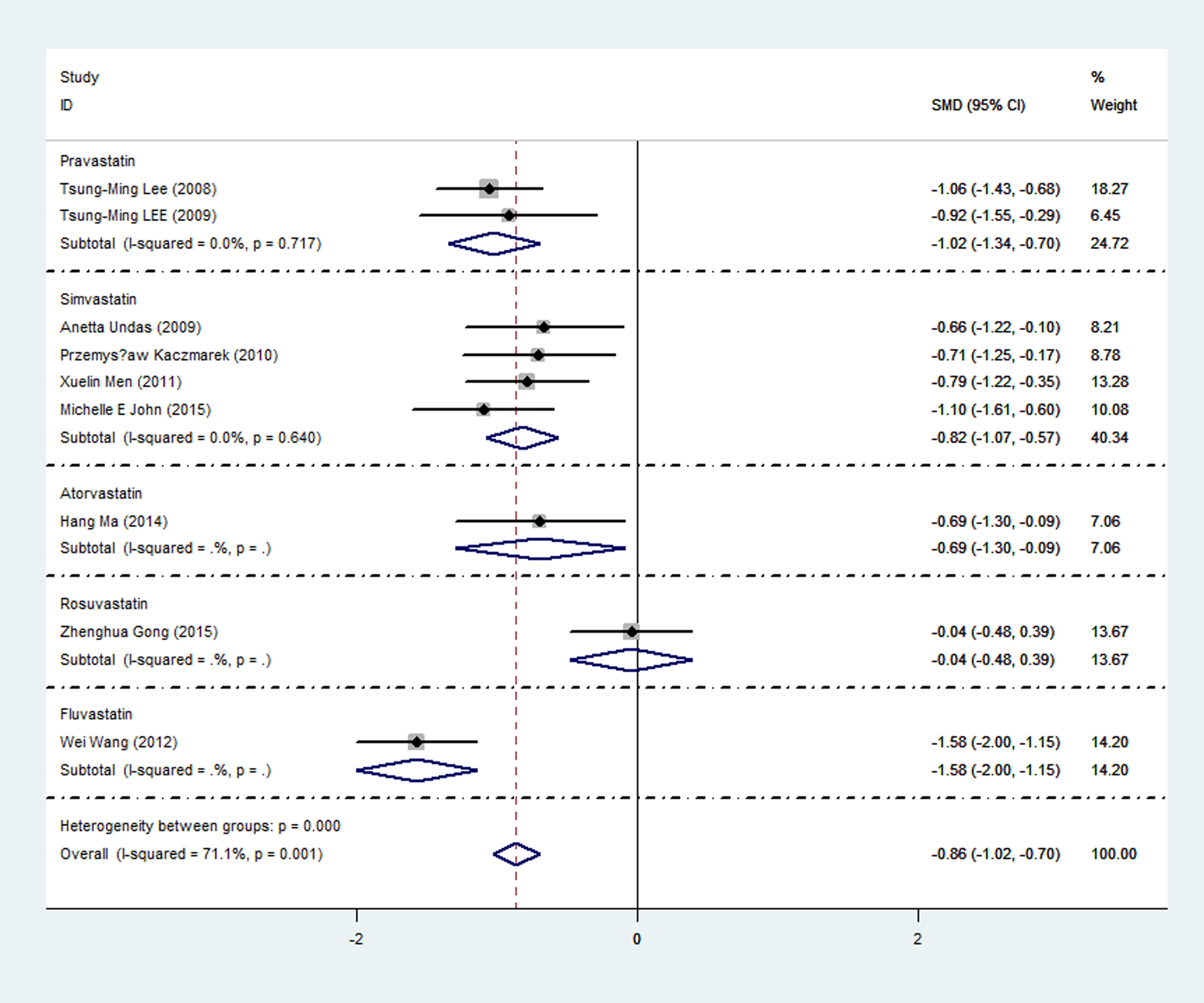

Supplement: Supplementary file 26 — Forest plot showing effect of statins on TC in COPD patients. (TIF 31508 kb) [file 12931_2019_984_MOESM26_ESM.tif]

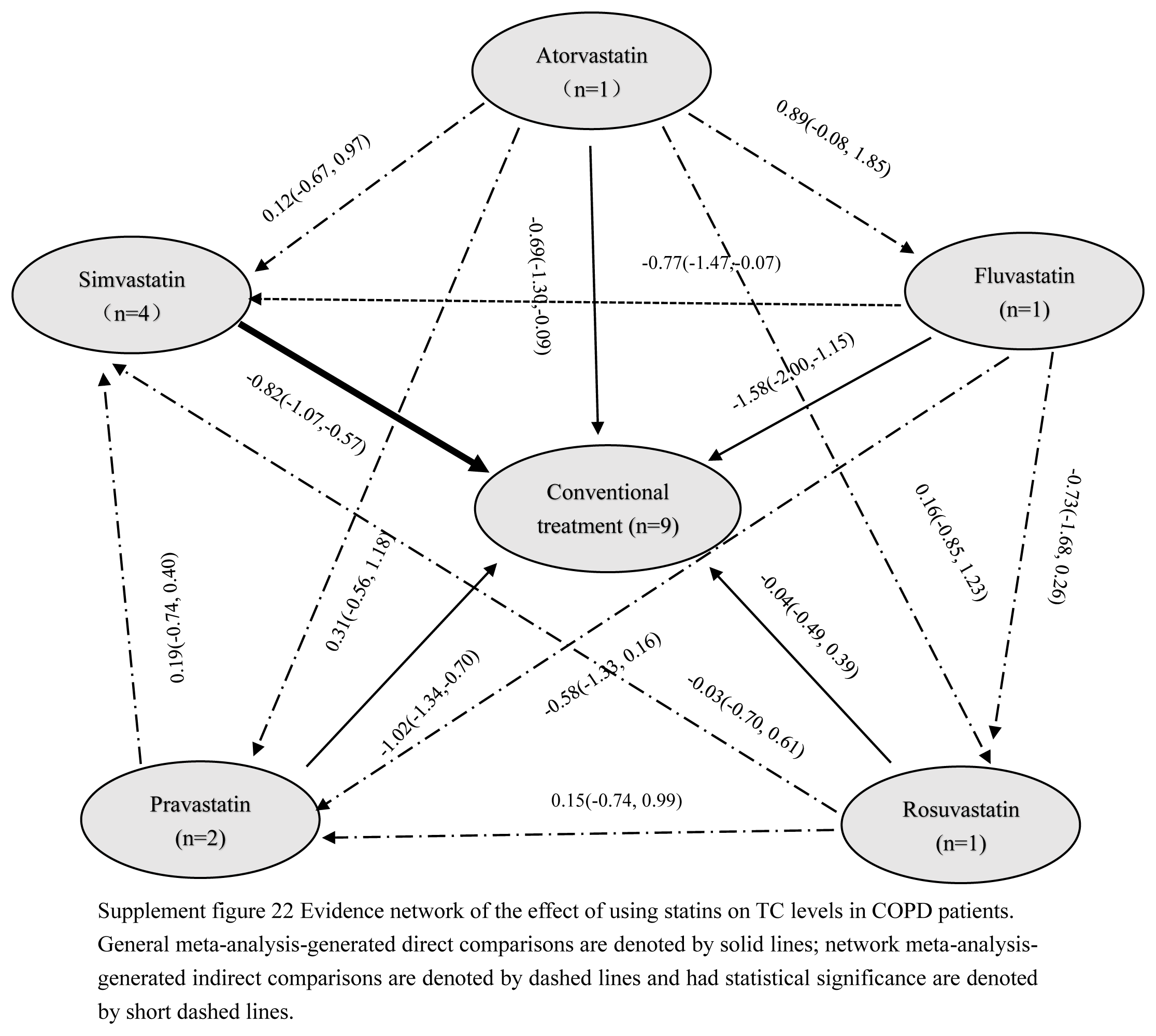

Supplement: Supplementary file 27 — Evidence network of the effect of using statins on TC levels in COPD patients. (TIF 13642 kb) [file 12931_2019_984_MOESM27_ESM.tif]

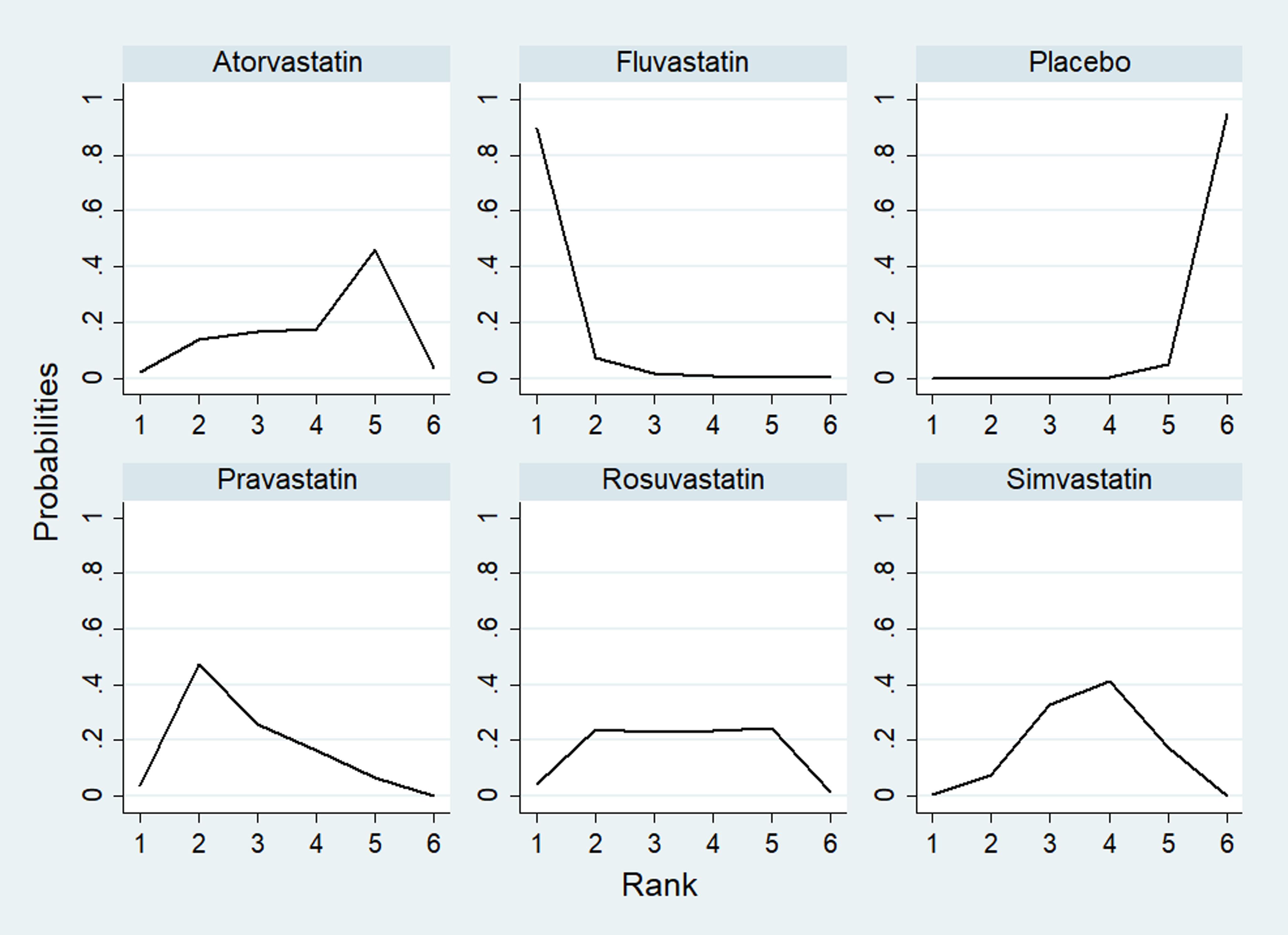

Supplement: Supplementary file 29 — Rank probability analysis of TC with using statins in COPD patients. (TIF 36014 kb) [file 12931_2019_984_MOESM29_ESM.tif]

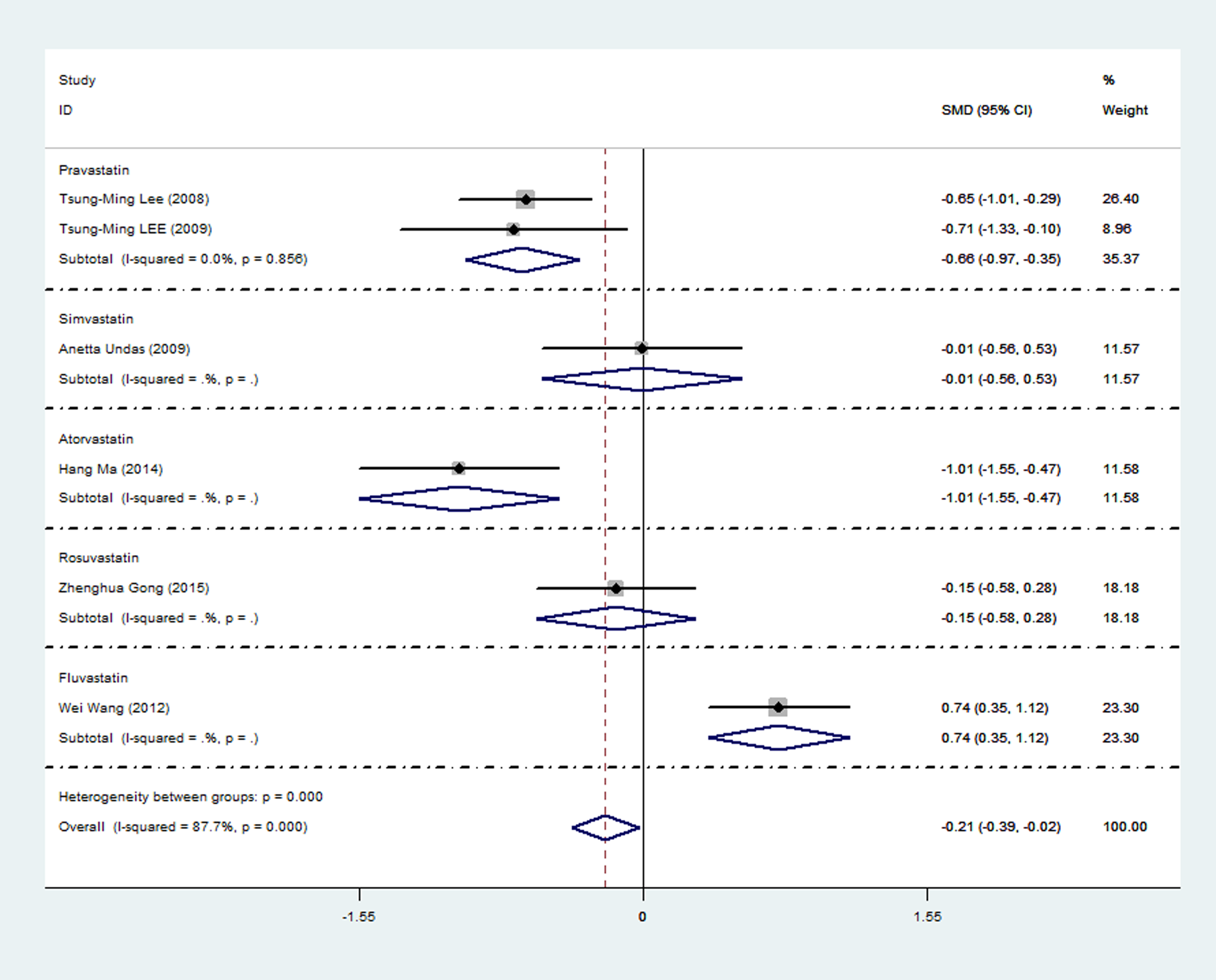

Supplement: Supplementary file 30 — Forest plot showing effect of statins on TG in COPD patients. (TIF 32559 kb) [file 12931_2019_984_MOESM30_ESM.tif]

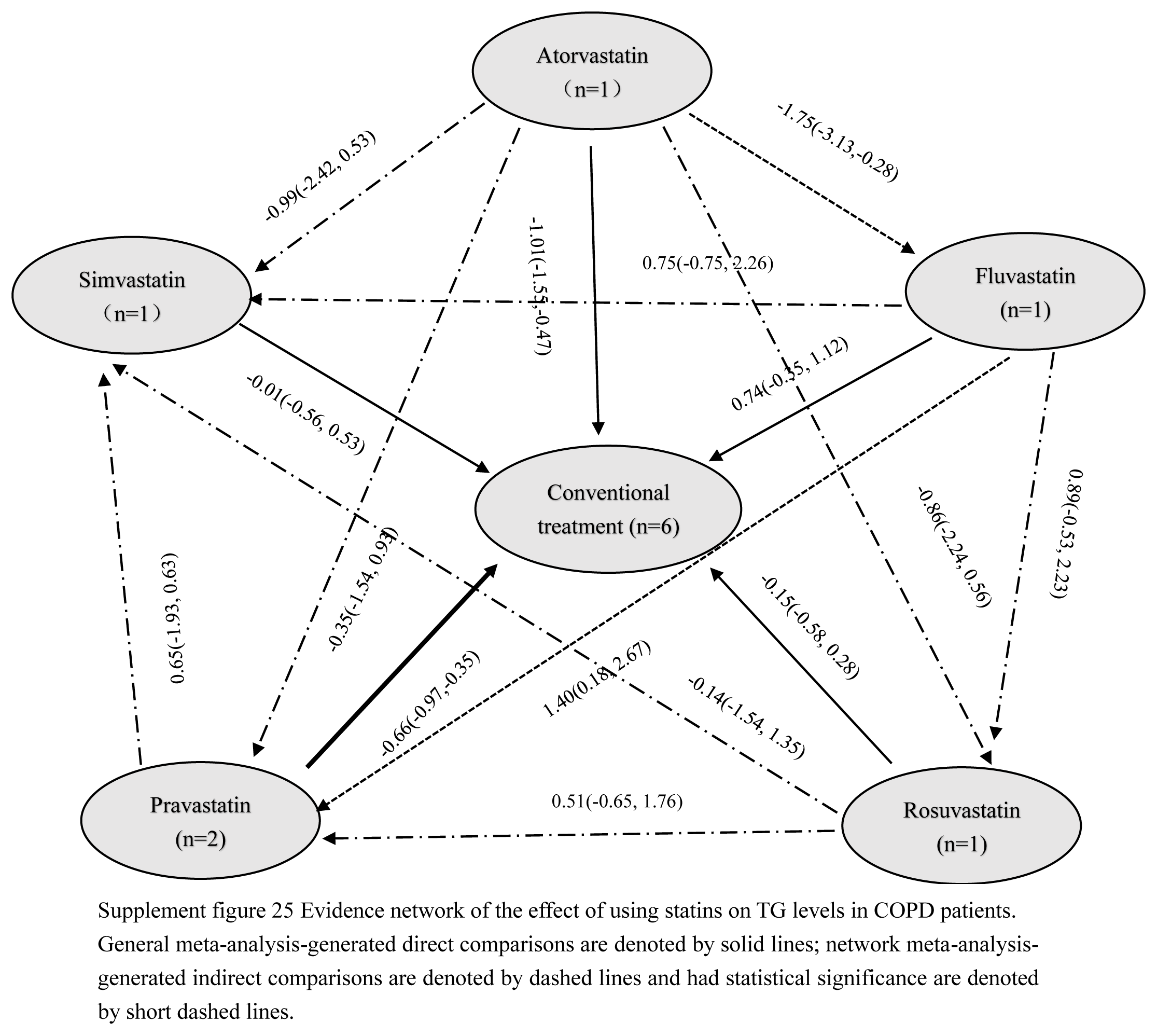

Supplement: Supplementary file 31 — Evidence network of the effect of using statins on TG levels in COPD patients. (TIF 13633 kb) [file 12931_2019_984_MOESM31_ESM.tif]

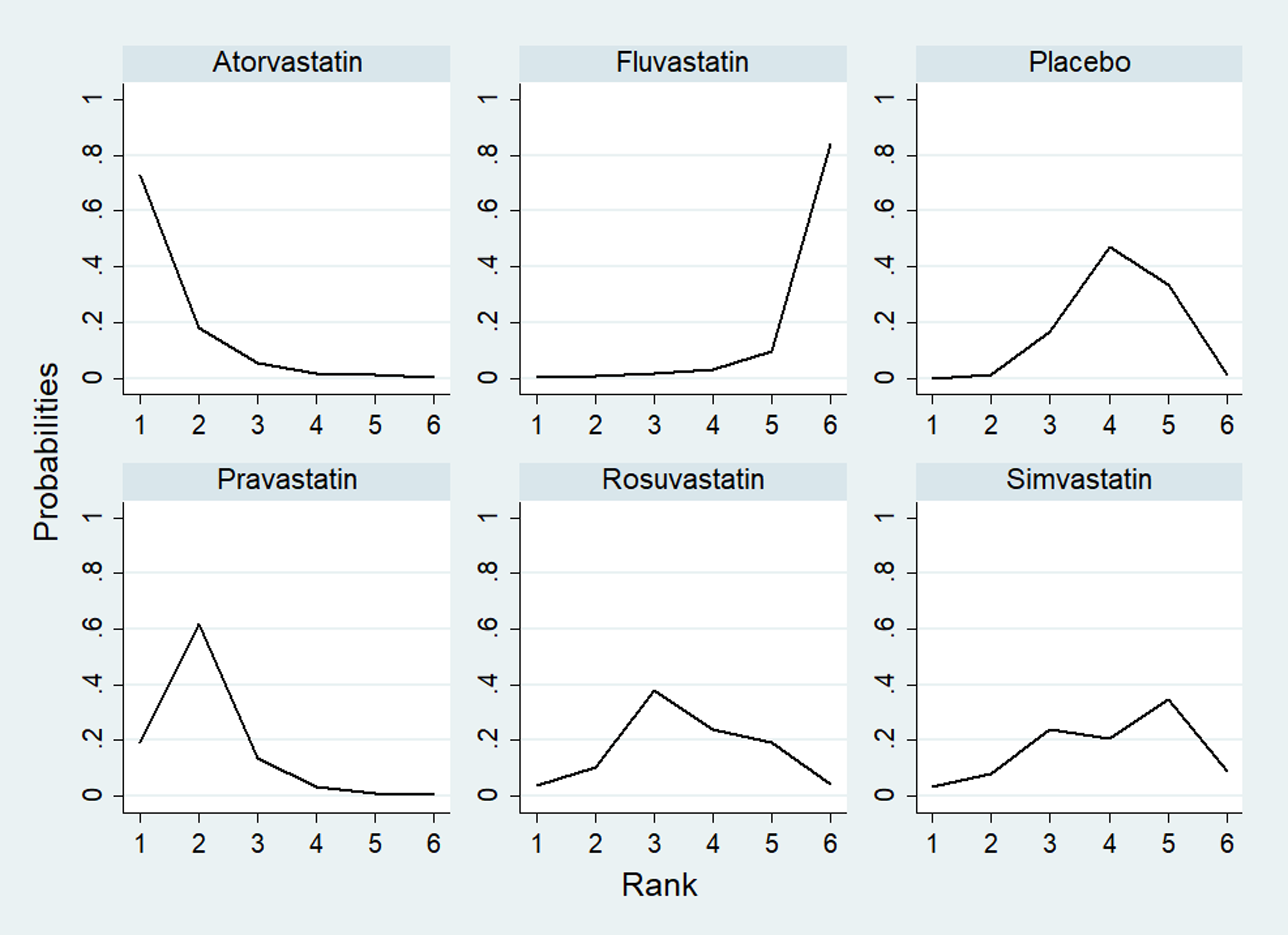

Supplement: Supplementary file 33 — Rank probability analysis of TG with using statins in COPD patients. (TIF 36014 kb) [file 12931_2019_984_MOESM33_ESM.tif]
