# Supplementary material for: Effectiveness of long-term using statins in COPD – a network meta-analysis
Source: Respir Res. 2019 Jan 23;20:17. doi: 10.1186/s12931-019-0984-3 (PMC6343315; doi:10.1186/s12931-019-0984-3)
Supplement: Supplementary file 7 — Rank probability analysis of IL-6 with using statins in COPD patients. (PDF 177 kb) [file 12931_2019_984_MOESM7_ESM.pdf]

Supplement table 1 Rank probability analysis of IL-6 with using statins in COPD patients

Treatment	SUCRA	sd	2.50%	median	97.50%
Atorvastatin	52.7	0.2066	0.2	0.6	1.0
Fluvastatin	25.5	0.2888	0.0	0.2	1.0
Rosuvastatin	70.7	0.2913	0.0	0.8	1.0
Pravastatin	58.3	0.3082	0.0	0.6	1.0
Simvastatin	79.9	0.1961	0.4	0.8	1.0
Conventional treatment	12.8	0.1305	0.0	0.2	0.4
