# Supplementary material for: Effectiveness of long-term using statins in COPD – a network meta-analysis
Source: Respir Res. 2019 Jan 23;20:17. doi: 10.1186/s12931-019-0984-3 (PMC6343315; doi:10.1186/s12931-019-0984-3)
Supplement: Supplementary file 12 — Rank probability analysis of TNF-α with using statins in COPD patients. (PDF 272 kb) [file 12931_2019_984_MOESM12_ESM.pdf]

Supplement table 2 Rank probability analysis of TNF-α with using statins in COPD patients

Treatment	SUCRA	sd	2.50%	median	97.50%
Atorvastatin	51.0	0.3195	0.0	0.5	1.0
Simvastatin	89.2	0.2080	0.5	1.0	1.0
Conventional treatment	9.8	0.1995	0.0	0.0	0.5
