# Supplementary material for: Effectiveness of long-term using statins in COPD – a network meta-analysis
Source: Respir Res. 2019 Jan 23;20:17. doi: 10.1186/s12931-019-0984-3 (PMC6343315; doi:10.1186/s12931-019-0984-3)
Supplement: Supplementary file 16 — Rank probability analysis of FEV1% with using statins in COPD patients. (PDF 177 kb) [file 12931_2019_984_MOESM16_ESM.pdf]

Supplement table 3 Rank probability analysis of FEV1% with using statins in COPD patients

Treatment	SUCRA	sd	2.50%	median	97.50%
Atorvastatin	17.0	0.1738	0.0	0.2	0.6
Fluvastatin	91.2	0.1717	0.4	1.0	1.0
Rosuvastatin	11.0	0.1563	0.0	0.0	0.6
Pravastatin	54.2	0.1607	0.2	0.6	0.8
Simvastatin	42.3	0.1631	0.0	0.4	0.8
Conventional treatment	84.0	0.0986	0.6	0.8	1.0
