# Supplementary material for: Effectiveness of long-term using statins in COPD – a network meta-analysis
Source: Respir Res. 2019 Jan 23;20:17. doi: 10.1186/s12931-019-0984-3 (PMC6343315; doi:10.1186/s12931-019-0984-3)
Supplement: Supplementary file 20 — Rank probability analysis of FEV1/FVC% with using statins in COPD patients. (PDF 177 kb) [file 12931_2019_984_MOESM20_ESM.pdf]

Supplement table 4 Rank probability analysis of FEV1/FVC% with using statins in COPD patients

Treatment	SUCRA	sd	2.50%	median	97.50%
Atorvastatin	19.4	0.2440	0.0	0.2	0.8
Fluvastatin	93.3	0.1677	0.4	1.0	1.0
Rosuvastatin	36.2	0.2795	0.0	0.4	1.0
Pravastatin	45.3	0.2488	0.0	0.4	1.0
Simvastatin	30.3	0.2191	0.0	0.2	0.8
Conventional treatment	75.6	0.1304	0.4	0.8	1.0
