# Supplementary material for: Effectiveness of long-term using statins in COPD – a network meta-analysis
Source: Respir Res. 2019 Jan 23;20:17. doi: 10.1186/s12931-019-0984-3 (PMC6343315; doi:10.1186/s12931-019-0984-3)
Supplement: Supplementary file 24 — Rank probability analysis of 6MW with using statins in COPD patients. (PDF 176 kb) [file 12931_2019_984_MOESM24_ESM.pdf]

Supplement table 5 Rank probability analysis of 6MW with using statins in COPD patients

Treatment	SUCRA	sd	2.50%	median	97.50%
Atorvastatin	30.5	0.2411	0.0	0.3	0.8
Rosuvastatin	46.6	0.4740	0.0	0.0	1.0
Pravastatin	43.1	0.2786	0.0	0.5	1.0
Simvastatin	47.8	0.4742	0.0	0.8	1.0
Conventional treatment	13.5	0.1839	0.0	0.0	0.5
