# Supplementary material for: Effectiveness of long-term using statins in COPD – a network meta-analysis
Source: Respir Res. 2019 Jan 23;20:17. doi: 10.1186/s12931-019-0984-3 (PMC6343315; doi:10.1186/s12931-019-0984-3)
Supplement: Supplementary file 28 — Rank probability analysis of TC with using statins in COPD patients. (PDF 177 kb) [file 12931_2019_984_MOESM28_ESM.pdf]

Supplement table 6 Rank probability analysis of TC with using statins in COPD patients

Treatment	SUCRA	sd	2.50%	median	97.50%
Atorvastatin	39.5	0.2460	0.0	0.4	0.8
Fluvastatin	96.9	0.1069	0.6	1.0	1.0
Rosuvastatin	51.1	0.2481	0.2	0.6	1.0
Pravastatin	65.1	0.2003	0.2	0.8	1.0
Simvastatin	46.4	0.1725	0.2	0.4	0.8
Conventional treatment	1.1	0.0472	0.0	0.0	0.2
