# Supplementary material for: Effectiveness of long-term using statins in COPD – a network meta-analysis
Source: Respir Res. 2019 Jan 23;20:17. doi: 10.1186/s12931-019-0984-3 (PMC6343315; doi:10.1186/s12931-019-0984-3)
Supplement: Supplementary file 32 — Rank probability analysis of TG with using statins in COPD patients. (PDF 177 kb) [file 12931_2019_984_MOESM32_ESM.pdf]

Supplement table 7 Rank probability analysis of TG with using statins in COPD patients

Treatment	SUCRA	sd	2.50%	median	97.50%
Atorvastatin	91.6	0.1689	0.4	1.0	1.0
Fluvastatin	5.3	0.1472	0.0	0.0	0.6
Rosuvastatin	48.5	0.2312	0.0	0.6	1.0
Pravastatin	78.7	0.1600	0.4	0.8	1.0
Simvastatin	39.4	0.2514	0.0	0.4	1.0
Conventional treatment	36.5	0.1510	0.2	0.4	0.6
